# Oligomerization of Lrrk controls actin severing and α-synuclein neurotoxicity in vivo

**DOI:** 10.1186/s13024-021-00454-3

**Published:** 2021-05-24

**Authors:** Souvarish Sarkar, Farah Bardai, Abby L. Olsen, Kelly M. Lohr, Ying-Yi Zhang, Mel B. Feany

**Affiliations:** 1 Department of Pathology, Brigham and Women’s Hospital, Harvard Medical School, Massachusetts Boston, USA; 2 Department of Neurology, Brigham and Women’s Hospital, Harvard Medical School, Massachusetts Boston, USA; 3 Department of Medicine, Brigham and Women’s Hospital, Harvard Medical School, Massachusetts Boston, USA

**Keywords:** Parkinson’s disease, LRRK2, α-synuclein, F-actin, Drp1, Mitochondria, Drosophila, Genetics, Dopamine neuron, Bioenergetics

## Abstract

**Background:**

Mutations in LRRK2 are the most common cause of familial Parkinson’s disease and typically cause disease in the context of abnormal aggregation and deposition of α-synuclein within affected brain tissue.

**Methods:**

We combine genetic analysis of Lrrk-associated toxicity in a penetrant *Drosophila* model of wild type human α-synuclein neurotoxicity with biochemical analyses and modeling of LRRK2 toxicity in human neurons and transgenic mouse models.

**Results:**

We demonstrate that Lrrk and α-synuclein interact to promote neuronal degeneration through convergent effects on the actin cytoskeleton and downstream dysregulation of mitochondrial dynamics and function. We find specifically that monomers and dimers of Lrrk efficiently sever actin and promote normal actin dynamics in vivo. Oligomerization of Lrrk, which is promoted by dominant Parkinson’s disease-causing mutations, reduces actin severing activity in vitro and promotes excess stabilization of F-actin in vivo. Importantly, a clinically protective Lrrk mutant reduces oligomerization and α-synuclein neurotoxicity.

**Conclusions:**

Our findings provide a specific mechanistic link between two key molecules in the pathogenesis of Parkinson’s disease, α-synuclein and LRRK2, and suggest potential new approaches for therapy development.

**Supplementary Information:**

The online version contains supplementary material available at 10.1186/s13024-021-00454-3.

## Background

Parkinson’s disease is the second most common neurodegenerative disorder, after Alzheimer’s disease, and is the most common neurodegenerative movement disorder. Despite years of investigation and important advances in understanding the neuroanatomy, neuropathology, and, more recently, genetics of Parkinson’s disease, treatment for the inexorably progressive and severe disorder remain symptomatic [1, 2]. Mutations in the gene encoding α-synuclein, including both point mutations and duplications and triplications, produce penetrant familial forms of Parkinson’s disease [3–9]. Further, aggregation and deposition of α-synuclein into proteinaceous Lewy inclusions in neurons and glia are the pathological hallmarks of Parkinson’s disease [10, 11] and related neurodegenerative disorders, collectively termed α-synucleinopathies. LRRK2, Leucine-rich repeat kinase 2, mutations are the most common cause of familial Parkinson’s disease and are an important risk factor for apparently sporadic disease. Thus, there has been intensive effort to delineate the cell biological mechanisms connecting α-synuclein and LRRK2.

α-synuclein is a small, 14 kDa, natively unstructured protein localized primarily to synaptic vesicles with an as yet incompletely understood role in regulating synaptic neurotransmitter release [12–14]. LRRK2, in contrast, is a large, approximately 250 kDa, member of the ROCO family of G-proteins. ROCO proteins contain a tandem Roc (Ras of complex proteins) GTPase domain and COR (C terminal of Roc) dimerization domain. In addition to the RocCOR domain, the ROCO family members LRRK1 and LRRK2 have a leucine-rich repeat (LRR) N-terminal and both kinase and WD40 domains C-terminal to the RocCOR [15]. LRRK2 localizes to the cytosol and multiple membrane compartments. A number of binding partners and molecular and cellular functions have been described for LRRK2, including roles in vesicle trafficking [16–23], autophagy [24, 25], miRNA and translational regulation [26, 27], and nucleoskeletal organization [28]. The way in which these LRRK2 binding proteins and cellular functions relate to disease pathogenesis and α-synuclein pathology, is not clearly understood. We have previously demonstrated that LRRK2 can sever purified actin filaments in vitro and promote normal actin dynamics in vivo [29]. These findings are intriguing in light of a critical role for disruption of spectrin-mediated F-actin dynamics we have recently described in a new *Drosophila* model of α-synucleinopathy [30]. Here we implicate abnormal regulation of the actin cytoskeleton and downstream mitochondrial dysfunction as convergent mediators of α-synuclein and Lrrk-associated neurotoxicity. We further identify oligomerization of LRRK as a key mediator of reduced actin severing activity linked to disease states.

## Methods

### Genetics

All *Drosophila* crosses and aging were performed at 25˚C. Biochemical and histopathological assays were typically performed on 10-day-old flies, with exceptions as noted in the figure legends. The human *QUAS-wild type α-synuclein* line has been described previously [30]. Experimental and control genotypes typically contained both the *nSyb-QF2* and *nSyb-GAL4* drivers, with the exception of experiments with *Lrrke*^*03680*^ homozygous mutants, where *QUAS-wild type α-synuclein, Lrrke*^*03680*^/*nSyb-GAL4, Lrrke*^*03680*^ transheterozygotes were used for most experiments. Additional details regarding specific genotypes are provided in the figure legends. The following *Drosophila* stocks were kindly provided by the indicated investigators: *Lrrke*^*03680*^, *UAS-Lrrk RNAi*, *UAS-Lrrk*^*wt*^, *UAS-Lrrk*^*R1069C*^, *UAS-Lrrk*^*I1915T*^ and *UAS-Lrrk*^*Y1383C*^, *UAS-Lrrk RNAi*, *UAS-Lrrk-3KD, UAS-LRRK2*, Dr. Bingwei Lu; *UAS-Lrrk*^*G1914S*^, Dr. Ming Guo; *Lrrk*^*HA*^ knockin animals, Dr. Patrick Verstreken; *FLAG-FlAsH-HA-Drp1*, Dr. Hugo Bellen; *UAS-mito-GFP*, Dr. Thomas Schwarz; *nSyb-QF2*, Dr. Christopher Potter. *nSyb-GAL4* and *UAS-GFP-mCherry-Atg8a* were obtained from the Bloomington *Drosophila* Stock Center.

Heterozygous LRRK2-G2019S (C57BL/6-Lrrk2tm4.1Arte) and age- and sex-matched control (C57BL/6) mice were obtained from Taconic Biosciences. LRRK2-KO (C57BL/6-Lrrk2tm1.1Mjff/J) mice were obtained from the Jackson Laboratory. Animals were sacrificed at 10–12 weeks of age. Mice were housed and treated in accordance with the NIH Guide for the Care and Use of Laboratory Animals. All animal procedures were approved and performed in accordance with the Brigham and Women’s Hospital Institutional Animal Care and Use Committee. Mice were maintained in a pathogen-free facility on a 12-hour light/dark cycle with water and food provided ad libitum. For all *Drosophila* and mouse experiments equivalent numbers of male and female animals were used.

### Cell culture

Heterozygous LRRK-G2019S and isogenic control iPS cells were obtained from Axol and differentiated into neurons following manufacturer’s recommendations and reagents. Briefly, cell culture plates were coated with SureBond, and cells were plated 24 h later coating in plating medium. At 24 h post plating, half of the media was changed to neural maintenance media. The following day all culture media was replaced with neural differentiation media. This medium was changed every 3 days. Ten days post differentiation experiments were performed. Incubators were maintained at 37 °C and 5 % CO2.

### Histology and immunostaining

*Drosophila* heads were fixed in formalin and embedded in paraffin. Serial frontal Sec. (2 or 4 μm) of the entire brain were prepared and mounted on glass slides. To assess neuronal density, sections were stained with hematoxylin and imaged using SPOT software. The number of anterior medulla neurons were counted using the cell counter plugin in ImageJ and the number of neurons per 1,000 µm^2^ are presented in the figures.

For immunostaining on paraffin sections, antigen retrieval was performed by microwaving in sodium citrate buffer, pH 6.0, for 15 min. Slides were then blocked in 2 % milk in PBS with 0.3 % Triton X-100, followed by overnight incubation with primary antibodies at room temperature. Primary antibodies were used at the indicated concentrations: mouse monoclonal actin (JLA20), 1:500, Developmental Studies Hybridoma Bank; rabbit polyclonal actin (A2066), 1:1000, Sigma; HA-11, 1:100, Covance; GFP (N86/8), 1:1000, NeuroMab; GFP (ab290), 1:2,000, Abcam; tyrosine hydroxylase, 1:200-1:500, Immunostar; GABARAP (E1J4E, endogenous Atg8a), 1:1,000–1:2,000, Cell Signaling; p62 (ref(2)P), 1:1,000–5,000; ß-tubulin III (TuJ-1), 1:1,000, Biolegend. After three washes with PBS-Triton, slides were incubated with appropriate secondary antibodies coupled to Alexa Fluor 488, Alexa Fluor 555 or Alexa Fluor 647. Rabbit polyclonal antibodies directed against *Drosophila* p62 (ref(2)P) were created by Covance using the peptide sequence PRTEDPVTTPRSTQ. Quantification of autophagosomal and lysosomal markers, as well as dopaminergic neurons were performed by immunostaining with biotinylated secondary antibodies, followed by avidin–biotin–peroxidase complex (Vectastain Elite; Vector Laboratories), as previously described [31, 32]. The number of tyrosine hydroxylase-positive medulla neurons were counted using the cell counter plugin in ImageJ and the number of neurons per 10,000 µm^2^ are presented in the figures. The number of Atg8a-positive puncta in the anterior medulla per 10,000 µm^2^ and the number of p62-positive puncta in one entire medulla are presented in Fig. 7. F-actin was stained in paraformaldehyde-fixed whole mount brains by incubating with Acti-Stain 555 phalloidin for one hour at a dilution of 1:500 in PBS with 0.3 % Triton. Brains were then washed 3 times in PBS for 60 min each, mounted and imaged using confocal microscopy. F-actin was stained in paraformaldehyde-fixed 15 μm mouse brain sections and paraformaldehyde-fixed human neurons at a dilution of 1:1,000.

### Confocal microscopy and image analysis

Images were obtained on a Zeiss LSM 800 confocal microscope. For Drp1 localization to mitochondria, images were collected using and processed using AIRYSCAN. Each image represents a Z-stack of at least 8 images, each with a mean thickness of 0.2 μm. All images were assessed for saturation, and a maximal projection of the 3D image was generated. For the Pearson’s coefficient calculation, a region of interest (ROI) was drawn around individual cells, and each ROI was subjected to a colocalization analysis using the ImageJ macro Coloc2. Each ROI was thresholded individually to avoid image saturation. The Pearson coefficient was obtained as an output of colocalization from Coloc2. The Pearson coefficient was calculated for 50–60 ROIs across six different biological replicates. The average Pearson coefficient value for each genotype was used for analysis. The number of actin-rich rods was determined by counting all rod-shaped or round structures over 3 microns in size that stained for actin and the total number of rods in a standardized section of the anterior medulla (approximately 30,000 µm^2^) are presented in the figures. For the quantification of fluorescence, average pixel density from two-dimensional projections of z-stacks for the entire brain was computed using ImageJ. GFP-mCherry-Atg8a structures were analyzed as previously described [33]. Briefly, images were opened in ImageJ and the intensity of mCherry and GFP was measured in 50 regions of interest (ROI) from each animal. For assessment of fluorescence, samples were processed simultaneously using the same acquisition parameters.

###  Gel electrophoresis and immunoblotting

For denaturing polyacrylamide gel electrophoresis, *Drosophila* brains were homogenized in 2X Laemmli sample buffer and the resulting homogenates analyzed on 4–20 % precast gels (Bio-Rad) and immunoblotted according to standard protocols. For native gel electrophoresis, native sample buffer (62.5 mM Tris-HCL, pH 6.8, 25 % glycerol, 1 % bromophenol blue), running buffer (25 mM Tris, 192 mM glycine, and 7.5 % precast gels (Bio-Rad) were used. Purified LRRK2 proteins (75 ng; Life Technologies, Creative Biomart) were incubated in for one hour at room temperature before electrophoresis. All blots were repeated at least three times with similar results. Images of representative blots are shown in the figures. Primary antibodies were used at the indicated concentrations: α-synuclein (H3C), 1:350,000, Developmental Studies Hybridoma Bank; LRRK2 (N214A/34), 1:200, NeuroMab; GAPDH, 1:10,000, Abcam; HA-11, 1:2,000, Covance. Silver staining was performed using the Thermo Scientific Pierce Silver Stain Kit (Cat # 24,162) following the manufacturer’s protocol.

### Metabolic analysis

The oxygen consumption rate (OCR) and extracellular acidification rate (ECAR) were measured using a Seahorse XFe96 Analyzer (Agilent Technologies) following the procedures recommended by the manufacturer. Briefly, for all the experiments, brains from 10-day-old flies were dissected and plated at one brain per well in XF96-well culture microplates plates and metabolic parameters assayed as described [34]. For iPS cells, approximately 40,000 cells were plated per well in XF96-well culture microplates plates. Basal respiration was recorded for six cycles, following which 1 µM rotenone was injected to inhibit mitochondrial respiration, and OCR and ECAR was recorded for four more cycles. Values were normalized to DNA content using a CyQUANT assay (ThermoFisher) following the manufacturer’s protocol.

### Size exclusion chromatography

Recombinant LRRK2 proteins (Life Technologies) were diluted in PBS at 0.25 mg/ml and incubated at 22˚C for 1 h. The solution (0.2 ml) was immediately loaded onto a Superdex 200 10/300 GL column (GE Healthcare) equilibrated with SEC buffer (20 mM Tris HCl, 150 mM NaCl, pH 7.5). The column was operated by NGC chromatography system (BioRad) and protein was eluted with an isocratic flow at 0.2 ml/min. Fractions (0.25 ml) were collected and combined into putative monomer, dimer and oligomer pools based on the calibration of the column and immunoblotting for LRRK2. Fractions containing monomers, dimers and oligomers of LRRK2 were used for further experiments.

### Actin depolymerization assay

Actin depolymerization assays were performed using the fluorescent form of the Actin Polymerization Biochem Kit from Cytoskeleton Inc. (Cat. #BK003), as described by the manufacturer. Pyrene-labeled actin was polymerized at room temperature for 1 h in actin polymerization buffer (containing 2 mM MgCl_2_ and 2 mM ATP) and then mixed either with buffer or with human recombinant LRRK2, LRRK2-G2019S, or a 1:1 mixture of the two. Actin was used at a final concentration of 4 µM and the LRRK2 proteins at 1 nM. The samples were read every 30 s in a plate reader at the excitation/emission wavelengths of 350/410 for 1 h. The experiment was repeated three times with two technical replicate each time per sample. The values were normalized to 0 at the starting time. Each data point represents the mean of three separate experiments.

### Actin severing assay

Actin (4 µM) was polymerized in actin polymerization buffer (containing 2 mM MgCl_2_ and 2 mM ATP) at room temperature for 1 h. Polymerized actin was incubated with LRRK2, LRRK2-GS or mix (1 nM final concentration) for 2 min. Fluorescently-labeled phalloidin (Acti-stain 555) was added to a final concentration of 2 µM, and the samples were diluted 50-fold with PBS. 2 µl of each sample was adsorbed on coverslips coated with 0.01 % poly-L-lysine and imaged using a fluorescence microscope. The filament lengths were quantified using ImageJ software by freehand drawing tool. Three different randomly selected areas were quantified for each sample with at least 10 filaments measured per area. The experiment was repeated three times.

### Quantitative real-time PCR

For reverse transcription, RNA was extracted from 4 fly heads using QIAzol (QIAGEN). 1 µg of RNA was reverse transcribed using the Applied Biosystems High-Capacity cDNA Reverse Transcription Kit using the manufacturer’s protocol. SYBR Green (Applied Biosystems) based qPCR was performed on an Applied Biosystems QuantStudio 6 Flex Real-Time PCR System. Primer sequences for Lrrk were CCGCTTGTTCCGTTGTTGTG (forward) and ATCTTTCCTGCAATTTCGC (reverse). Primer sequences for *RpL32*, used as internal control, were GACCATCCGCCCAGCATAC (forward) and CGGCGACGCACTCTGTT (reverse). The fold change in gene expression was determined by the ΔΔC_t_ method, where C_t_ is the threshold value.

### Statistical analysis

All reported n values are biological replicates. The sample sizes used were similar to the ones reported in previous publications [35, 36]. For *Drosophila* immunostaining, genetic reporter and dye labeling experiments the sample size was 6 per genotype and time point. Western blot quantifications were performed on at least three independent blots. The sample sizes for mouse experiments were determined by power analyses. For the histological studies we determined that a sample size of 5 is sufficient to detect 20 % difference between control and experimental genotypes with a power of 80 %. Exact sample size for each experiment is provided in the figure legends. Data collection and analysis in mouse experiments were performed blinded to the conditions of the experiment. Data analysis on *Drosophila* sections was also performed blinded. Statistical analysis was performed using one-way ANOVA and multiple comparisons among the datasets were performed. Variance was similar between groups compared.

## Results

### Lrrk promotes α-synuclein neurotoxicity in vivo

**Fig. 1:**
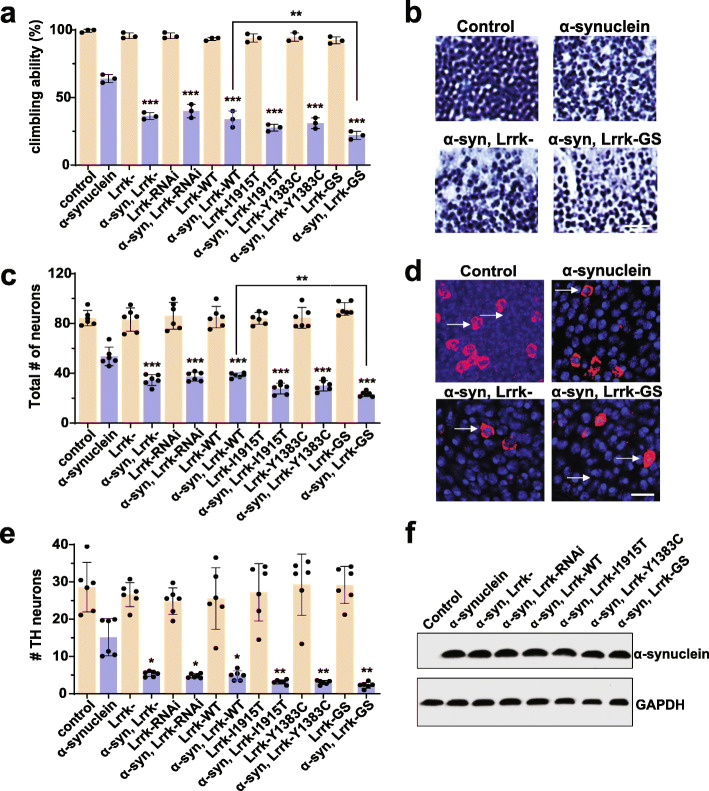
Modulation of Lrrk enhances α-synuclein neurotoxicity in *Drosophila*. **a**, Increasing or decreasing *Drosophila* Lrrk worsens the locomotor climbing defect induced by the expression of transgenic human wild type human α-synuclein, which is further enhanced by expressing mutant forms of Lrrk engineered to mimic Parkinson’s disease mutations. **b**, Altering Lrrk levels further enhances α-synuclein-mediated loss of hematoxylin-stained neurons in the anterior medulla, as quantified in (**c**) when compared to flies expressing α-synuclein alone. Scale bar represents 10 µm in (**b**). **d**, Increasing or decreasing Lrrk levels also further decreases the α-synuclein-induced loss of tyrosine hydroxylase-positive neurons in the anterior medulla (arrows), as quantified in (**e**) when compared to flies expressing α-synuclein alone. Scale bar represents 5 µm in (**d**). **f**, Western blotting reveals no change in α-synuclein levels with manipulation of Lrrk. The blot is reprobed for GAPDH to illustrate equivalent protein loading. Lrrk-: *Lrrk*^*e03680*^. Lrrk-WT: wild type Lrrk overexpression. Y1383C, I1915T and GS are Lrrk mutants homologous to Parkinson’s disease-associated human LRRK2 mutants Y1699C, I2020T, and G2019S respectively. *n*=6 per genotype. Data are represented as mean ± SD. *p<0.05, **p<0.01, ***p<0.005, ANOVA with Bonferroni post-test analysis. Control is *nSyb-GAL4, nSybQF2/+.* Flies are 10 days old in (**a-d**) and 1 day old in (**f**)

*Drosophila* contain a single ortholog of LRRK1 and LRRK2. Further simplifying genetic analysis, *Lrrk* protein null mutants are viable [26]. We have taken advantage of these genetic characteristics and a newly described *Drosophila* model of α-synucleinopathy [30] to assess the role of Lrrk loss and gain of function in mediating α-synuclein neurotoxicity. Our α-synucleinopathy model is based on expression of wild type human α-synuclein in a pan-neuronal pattern using the *nSyb-QF2* driver and the QF2 bipartite expression system [37, 38]. α-synuclein transgenic flies display accelerated age-dependent locomotor decline, degeneration of dopaminergic and non-dopaminergic neurons, and robust α-synuclein aggregation [30]. When we reduced Lrrk function using a protein null allele or transgenic RNAi (driven by *nSyb-GAL4*) in α-synuclein transgenic flies, we observed significant enhancement of locomotor dysfunction (Fig. 1a) and increased loss of both dopaminergic and non-dopaminergic neurons (Fig. 1b-e, arrows) in the anterior medulla, a site of strong expression with the *nSyb-QF2* and *nSyb-GAL4* drivers.

We next tested the effect of overexpressing wild type fly Lrrk on α-synuclein neurotoxicity. Interestingly, we observed enhancement of locomotor defects (Fig. 1a) and neurodegeneration (Fig. 1c,e) with overexpression of wild type Lrrk as well as with knockdown or knockout of Lrrk. To probe the role of disease-associated mutations in LRRK2, we used flies expressing Lrrk with mutations homologous to Parkinson disease-associated mutations in human LRRK2 [26]. The human mutations modeled included the most common Parkinson’s disease-associated mutation in human LRRK2, G2019S (G1914S in *Drosophila*) and I2020T (11915T in *Drosophila*). The G2019S and I2020T mutations are both in the kinase domain. We have termed the G2019S/G1914S mutation “GS” throughout the manuscript for clarity. We also examined Y1699C (Y1383C in *Drosophila*) in the adjacent COR domain. Expression of disease-linked mutant forms of Lrrk was toxic, with a tendency for mutant Lrrk to be more toxic than wild type Lrrk as shown by climbing ability (Fig. 1a), total neuron number (Fig. 1b,c), and dopaminergic neuron number (Fig. 1d,e). Reducing or increasing Lrrk expression in the absence of human α-synuclein was not toxic. Dopaminergic neuronal loss has previously been reported with expression of human LRRK2 in transgenic *Drosophila *[39, 40], although in flies older than the 10-day-old animals assessed here. Importantly, manipulation of Lrrk did not alter the levels of transgenic α-synuclein (Fig. 1f). The transgenes used mediate expression of similar levels of wild type and mutant Lrrk by western blot analysis [26, 29].

**Fig. 2:**
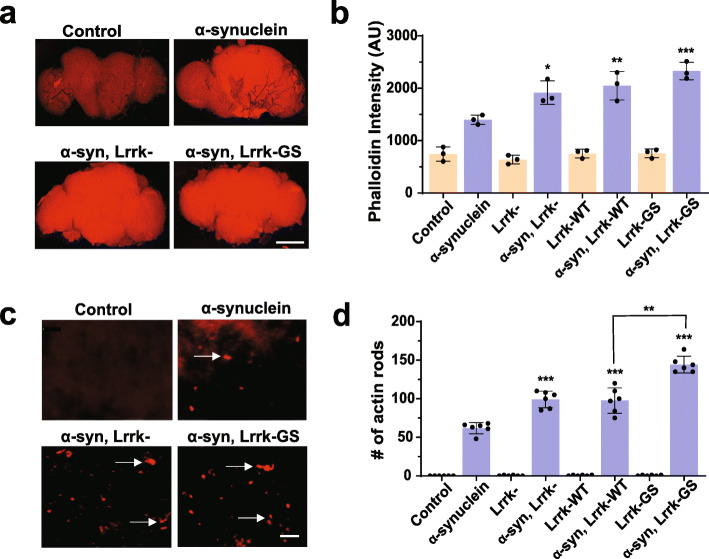
Manipulation of Lrrk enhances actin cytoskeletal pathology induced by α-synuclein. **a**, F-actin staining with fluorescent phalloidin in whole mount brains from flies expressing human α-synuclein shows increased F-actin compared to controls. F-actin is further increased when Lrrk protein is removed in homozygous *Lrrk*^*e03680*^ mutants, or mutant Lrrk-G1914S (Lrrk-GS), homologous to the human G2019S LRRK2 mutant, is expressed, as quantified in (**b**) when compared to flies expressing α-synuclein alone. Scale bar in (**a**) represents 75 µm. **c**, Immunofluorescent staining with an antibody to actin demonstrates rod-like inclusions when human α-synuclein is expressed in the fly brain. Numbers of rods are increased when Lrrk is removed or Lrrk-GS is expressed (arrows), as quantified in (**d**) when compared to flies expressing α-synuclein alone. Scale bar in (**c**) represents 15 µm. *n*=6 per genotype. Data are represented as mean ± SD. *p<0.05, **p<0.01,***p<0.005, ANOVA with Bonferroni post-test analysis. Control is *nSyb-GAL4, nSybQF2/+.* Flies are 10 days old

We have previously demonstrated increased F-actin levels and actin aggregation in α-synuclein transgenic flies [30]. We have additionally shown that Lrrk controls actin cytoskeletal dynamics [29]. To determine if manipulation of Lrrk can alter the actin cytoskeleton in α-synuclein transgenic flies, we first stained whole mount brain preparations with fluorescent phalloidin to assess F-actin levels. We found that reducing Lrrk levels in homozygous protein null *Lrrk* mutants significantly increased F-actin levels (Fig. 2a,b). Overexpression of *Drosophila* Lrrk carrying the G2019S homologous mutation (Lrrk-GS) also increased the levels of F-actin. Similarly, the number of rod-shaped actin aggregates in α-synuclein transgenic fly brains was increased by reducing Lrrk levels or expressing the Lrrk-GS mutant (Fig. 2c arrows, d).

### Oligomerization of LRRK2 controls actin dynamics

**Fig. 3:**
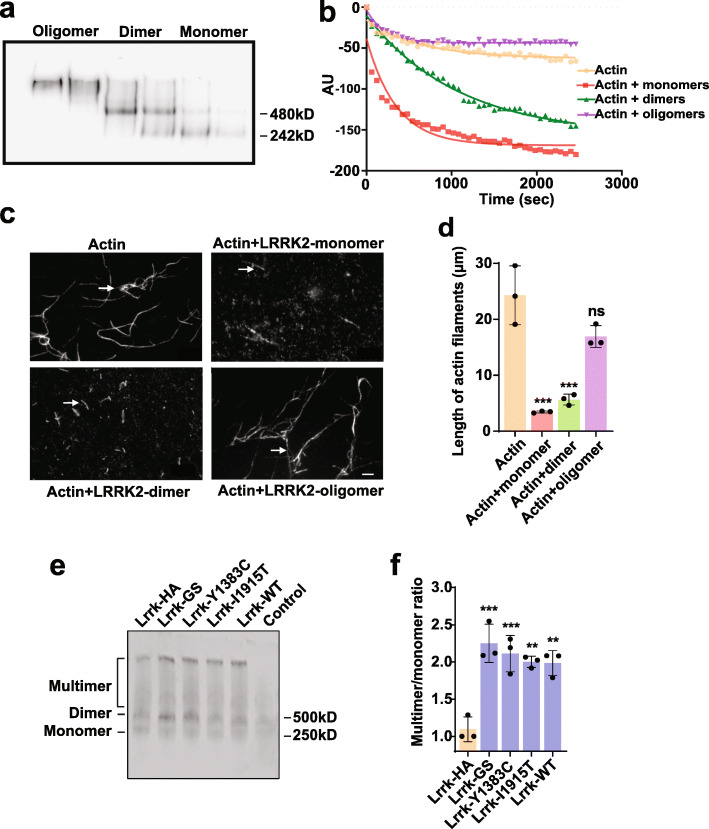
Lrrk enhances actin depolymerization by severing F-actin filaments in vitro and in vivo. **a**, Purified human LRRK2 protein was subjected to size exclusion chromatography following a one hour incubation to allow dimerization and oligomerization. Fractions containing putative monomer, dimer and oligomer pools were collected and confirmed by native gel electrophoresis and western blotting for LRRK2. **b**, Increased actin depolymerization mediated by monomers and dimers, but not oligomers of LRRK2 in a fluorescence-based pyrene actin depolymerization assay. *n*=3. **c**, Severing of fluorescently labeled actin filaments (arrows) by monomers and dimers of LRRK2, but not oligomers, in an in vitro assay, as quantified in (**d**). *n*=3. Scale bar in (**c**) represents 10 µm. **e**, Native western blotting of fly heads using endogenous Lrrk tagged with HA shows that multimer to monomer ratios in vivo, as quantified in (**f**) are increased in Lrrk mutants when compared to controls. *n*=3-6 per genotype. Data are represented as mean ± SD. *p<0.05, **p<0.01, ***p<0.005, ns, not significant, ­­ANOVA with Bonferroni post-test analysis. Control is *nSyb-GAL4/+.* Lrrk-HA is *Lrrk*^HA^, nSyb-GAL4/+. Flies are 10 days old

Having demonstrated a robust in vivo interaction between Lrrk and α-synuclein-mediated neurotoxicity and actin cytoskeletal stabilization, we next explored the underlying molecular mechanism. We have previously demonstrated that LRRK2 displays actin severing activity in vitro [29], but the molecular species mediating actin severing have not been defined. We therefore performed size exclusion chromatography on purified human LRRK2 protein following incubation of the protein for one hour at room temperature to allow formation of dimers and higher order oligomers (Fig. 3a). Fractions containing LRRK2 monomers, dimers or oligomers were then added to an in vitro fluorimeter-based depolymerization assay using pyrene labeled actin. Both monomers and dimers of LRRK2 promoted actin depolymerization, while oligomers were ineffective (Fig. 3b). To assess more directly the ability of LRRK2 species to sever actin, prepolymerized, fluorescently labeled actin filaments were incubated with chromatography fractions containing either LRRK2 monomers, dimers or oligomers of LRRK2 or without added LRRK2 and were then visualized with microscopy (Fig. 3c arrows). Similar to results from the pyrene-based depolymerization assay (Fig. 3a,b), monomers and dimers severed actin filaments, while oligomers did not show significant actin severing activity (Fig. 3d).

We then examined higher order Lrrk species in vivo using a *Drosophila* line in which the endogenous Lrrk protein is tagged with HA [41]. We performed native polyacrylamide gel electrophoresis followed by western blotting for HA in flies containing one copy of HA-tagged Lrrk and one copy of wild type or mutant Lrrk (Fig. 3e,f). Expression of Lrrk, including forms of Lrrk analogous to the human mutations Y1699C, I2020T and G2019S (Lrrk-I1915T, Lrrk-Y1383C and Lrrk-GS, respectively), promoted multimerization of Lrrk in vivo, consistent with their ability to increase F-actin levels (Fig. 2) and enhance neurotoxicity of α-synuclein (Fig. 1). In vitro aggregation assays followed by native polyacrylamide gel electrophoresis, silver staining and western blotting demonstrated increased oligomerization of LRRK2 when equimolar ratios of purified human wild type LRRK2 and mutant LRRK2 were incubated for one hour at room temperature to allow dimerization and multimerization (Supplementary Fig. 1a,b).

### Perturbed actin dynamics promotes mitochondrial dysfunction

**Fig. 4:**
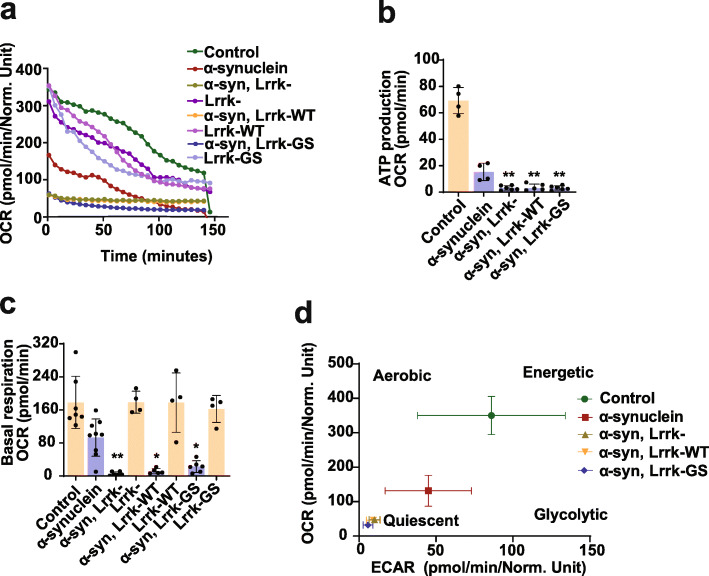
Lrrk promotes mitochondrial dysfunction and Drp1 mislocalization in α-synuclein transgenic flies.** a-d**, Metabolic profiling of whole brains in Seahorse XF 96-well culture microplates (Agilent) reveals decreased oxygen consumption rate (**a, **OCR), mitochondrial ATP production (**b**) and basal respiration (**c**) mediated by a-synuclein expression, which is worsened by Lrrk manipulation when compared to flies expressing α-synuclein alone. **d**, OCR and extracellular acidification rate (ECAR) from the same experiment plotted simultaneously reveal overall metabolic profiles for each genotype. *n*=4-9 per genotype. Data are represented as mean ± SD. *p<0.05, **p<0.01, ***p<0.005, ANOVA with Bonferroni post-test analysis. Control is *nSyb-GAL4, nSybQF2/+*. Flies are 10 days old

We next probed the cellular consequences of impaired F-actin dynamics in animals with altered Lrrk function. We have previously demonstrated that abnormal stabilization of the actin cytoskeleton promotes mitochondrial dysfunction through mislocalization of the critical fission protein Drp1 [29–31, 35]. We thus investigated mitochondrial function and morphology in α-synuclein transgenic flies following manipulation of Lrrk. A method has recently been described for assessment of metabolism in whole fly brains using the Agilent Seahorse XFe96 Analyzer [34], which we implemented in our α-synuclein transgenic animals. We observed a decrease in the oxygen consumption rate (OCR; Fig. 4a) and extracellular acidification rate (ECAR; Supplementary Fig. 2a) in flies expressing human α-synuclein in a pan-neuronal pattern. Further analysis revealed reduced mitochondrial ATP production and basal respiration mediated by α-synuclein expression (Fig. 4b,c). All of these metabolic parameters were worsened by removing Lrrk or increasing expression of either wild type or mutant Lrrk, as illustrated plotting the relative OCR by the ECAR acidification (Fig. 4d).

We then assessed mitochondrial morphology and the localization of Drp1 to mitochondria. We expressed mitochondrially-directed GFP (mito-GFP) to visualize mitochondria. We used a 9.35 kb genomic rescue strain that has an in-frame FLAG-FIAsH-HA tag after the start codon of Drp1, thus expressing tagged Drp1 under its endogenous promoter [35, 42] to visualize Drp1. The fission protein Drp1 is normally localized to round to modestly elongated mitochondria in the *Drosophila* brain (Supplementary Fig. 2b arrows, c). In contrast, as we have previously described [30], when F-actin cytoskeletal organization was disrupted by expression of human α-synuclein, Drp1 shifted to the cytoplasm and mitochondria became enlarged and displayed abnormal morphology. Removing Lrrk or expressing mutant Lrrk further exacerbated mitochondrial morphological abnormalities and Drp1 mislocalization (Supplementary Fig. 2b arrows, c). Drp1 protein levels remained unchanged in the heads of flies with Lrrk genetic manipulation (Supplementary Fig. 2d), demonstrating that the loss of Drp1 localization to the mitochondria was not due to a reduction in Drp1 levels.

### GTPase domain neuroprotective mutation reduces oligomerization and α-synuclein neurotoxicity

Since increased oligomerization of Lrrk correlates with enhanced neurotoxicity, we hypothesized that clinically-defined neuroprotective mutations in LRRK2 might decrease oligomerization. A number of studies have pointed to the GTPase activity of LRRK2 as a key mediator of dimer formation [43–47], making the human protective mutant LRRK2-R1398H in the Roc GTPase domain an attractive candidate modeling. We first confirmed that Lrrk containing a mutation (Lrrk-R1069C) homologous to the Parkinson’s disease-promoting Roc GTPase mutation LRRK2-R1441C enhanced the toxicity of transgenic human α-synuclein. Flies expressing Lrrk-R1069C with human α-synuclein had worsened locomotor function and enhanced degeneration of dopaminergic and nondopaminergic neurons (Supplementary Fig. 3a-e, arrows), and no change in the levels of transgenic human α-synuclein (Supplementary Fig. 3f). Similar to other pathogenic LRRK2 mutations studied here (Figs. 2 and 4), expression of Lrrk-R1069C with human α-synuclein promoted actin cytoskeletal stabilization as assessed by increased numbers of actin rods (Supplementary Fig. 3 g arrows, h), and enhanced mitochondrial bioenergetic defects (Supplementary Fig. 3i,j). Native polyacrylamide gel electrophoresis followed by western blotting demonstrated increased multimerization of the R1441C orthologous mutant protein in vivo (Supplementary Fig. 3k,l).

**Fig. 5:**
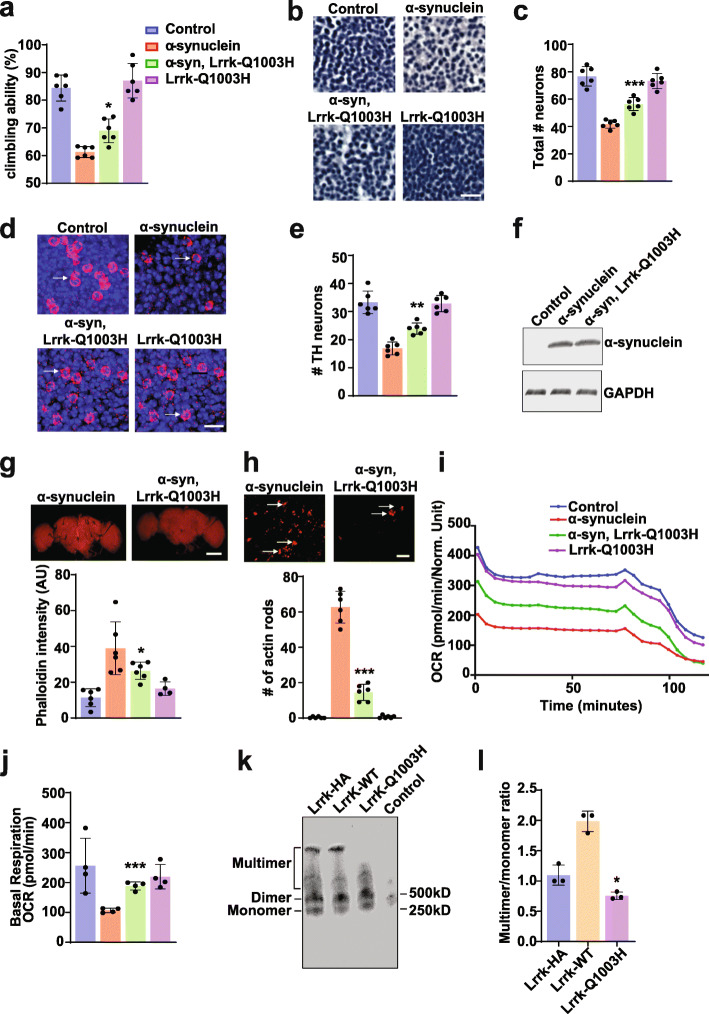
Expression of a clinically protective Lrrk mutant reduces α-synuclein neurotoxicity and decreases Lrrk oligomerization.** a**, Expression of Lrrk-Q1003H, analogous to the protective human LRRK2-R1398H variant, rescues the locomotor dysfunction produced by expression of α-synuclein. **b-c**, Lrrk-Q1003H suppresses the loss of medullary hematoxylin-stained neurons (**b**), as quantified in (**c**), and tyrosine hydroxylase immunostained neurons (**d **arrows), as quantified in (**e**) when compared to flies expressing α-synuclein alone. Scale bars represent 10 µm in (**b) **and 5 µm in (**d**). *n*=6 per genotype. **f**, Western blotting reveals no change in transgenic human α-synuclein levels with expression of Lrrk-Q1003H. The blot is reprobed for GAPDH to illustrate equivalent protein loading. **g,** Increased levels of F-actin as assayed by phalloidin staining on whole mount brains (top) and quantified (bottom) are reversed by expressing Lrrk-Q1003H. Scale bar in (**g**) represents 75 µm. **h, **Similarly, the formation of actin rods (top arrows) and quantified (bottom) is also rescued by expression of Lrrk-Q1003H when compared to flies expressing α-synuclein alone. Scale bar in (**h**) represents 15 µm. **i,j**, The α-synuclein induced decline in OCR is partially rescued by expression of Lrrk-Q1003H when compared to flies expressing α-synuclein alone. **k**, Native gel electrophoresis followed by western blotting (**k**) and quantitative analysis (**l**) demonstrates a significant reduction in the ratio of Lrrk multimer to monomer in flies expressing Lrrk-Q1003H compared to Lrrk-HA control flies. Data are represented as mean ± SD. *n*=3-6 per genotype. *p<0.05, ** *p*<0.01, ***p<0.005, ns, not significant, ANOVA with Bonferroni post-test analysis. Control is *nSyb-GAL4, nSybQF2/+* in (**a-j**). Control is *nSyb-GAL4/+* in (**k**). Lrrk-HA is* Lrrk*^HA^, nSyb-GAL4/+ in (**k,l**). Flies are 10 days old in (**a-e, g-l**) and 1 day old in (**f**)

We then proceeded to express a GTPase domain mutant, Lrrk-Q1003H, designed to mimic the human protective LRRK2-R1398H mutant. Expression of Lrrk-Q1003H with human α-synuclein was not toxic, but rather ameliorated neurotoxicity as assessed by climbing ability and degeneration of dopaminergic and nondopaminergic neurons (Fig. 5a-e, arrows). Expression of Lrrk-Q1003H mutant did not alter the level of transgenic human α-synuclein (Fig. 5f) and was expressed at levels comparable to the other forms of Lrrk protein studied (Supplementary Fig. 4a). To investigate the cell biological basis of Lrrk-Q1003H-mediated neuroprotection, we assessed the actin cytoskeleton and found partial normalization of F-actin as determined by fluorescent phalloidin staining on whole mount fly brain preparations (Fig. 5 g, Supplementary Fig. 4b) and reduction in the number of actin rods (Fig. 5 h arrows, Supplementary Fig. 4c). Metabolic function was also normalized with increased OCR and basal respiration compared to flies expressing human α-synuclein in the absence of transgenic Lrrk (Fig. 5i,j). We then used our tagged Lrrk allele to assess the propensity of Lrrk-Q1003H to multimerize in vivo. We found a statistically significant reduction in Lrrk multimers when Lrrk-Q1003H was expressed (Fig. 5k,l).

In contrast to GTPase activity, the kinase activity of LRRK2 has been well studied and linked to neurotoxicity, particularly in the LRRK2-G2019S mutant form of the protein. To investigate the role of Lrrk kinase activity in enhancement of α-synuclein neurotoxicity, we expressed a mutant form of *Drosophila* Lrrk carrying three point mutations (K178M, D1882A and D1912A, analogous to K1906M, D1994A and D2017 in human LRRK2; Lrrk-3KD), which target the ATP binding and active site of the enzyme and reduce kinase activity in vitro [26, 48]. Expression of Lrrk-3KD was less effective than wild type or disease-associated forms of Lrrk at enhancing α-synuclein induced locomotor dysfunction (Supplementary Fig. 5a, Fig. 1a), neurodegeneration (Supplementary Fig. 5b-e, Fig. 1b-e), actin cytoskeletal dysregulation (Supplementary Fig. 5 g,h, Fig. 2c,d) and mitochondrial dysfunction (Supplementary Fig. i,j, Fig. 4a-d). There was not a statistically significant change in multimeric Lrrk levels when Lrrk-3KD was expressed (Supplementary Fig. 5k-l) or change in the expression of transgenic human α-synuclein (Supplementary Fig. 5f). These findings are consistent with a contribution of kinase activity to the toxic effects of Lrrk expression in α-synuclein transgenic flies.

### LRRK2 controls actin cytoskeletal dynamics and mitochondrial function in mammalian neurons

**Fig. 6:**
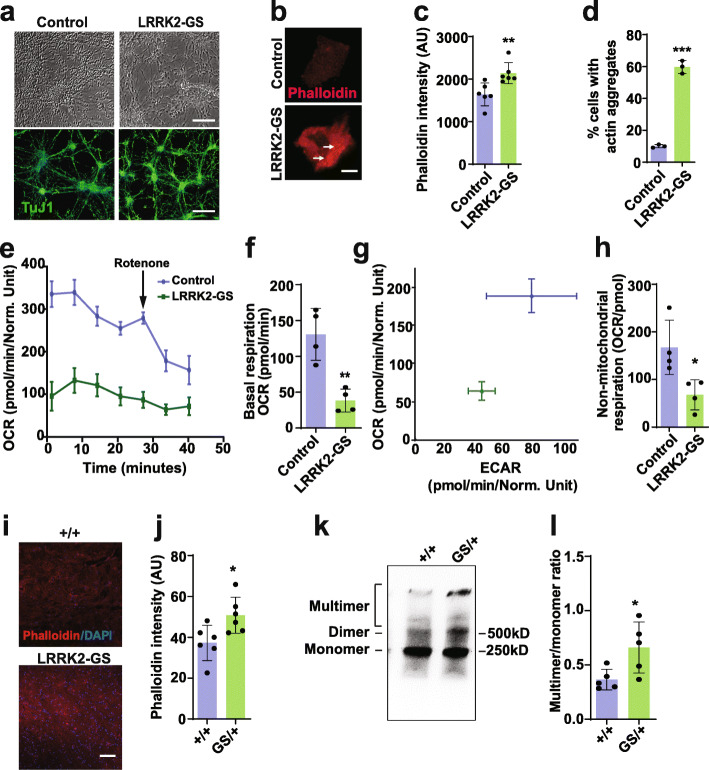
Mitochondrial dysfunction and actin cytoskeletal pathology in mammalian LRRK2-G2019S models.** a,** Morphology and expression of neuronal ß-tubulin (TuJ1) in cortical neurons derived from LRRK2-G2019S heterozygous iPSC and isogenic controls. Scale bars represent 40 µm in (**a**). **b-d**, LRRK2-G2019S heterozygous human neurons have increased F-actin as assessed by staining with fluorescent phalloidin (**b**), as well as numerous actin aggregates (arrows) as quantified in (**c,d**). Scale bar in (**b**) represents 15 µm. *n*=6 in (**c**), *n*=3 in (**d**). **e-h**, Metabolic profiling of cortical neurons derived from LRRK2-G2019S heterozygous iPSC and isogenic controls in Seahorse XF 96-well culture microplates reveals decreased oxygen consumption rate (**e, **OCR), basal respiration (**f**), the energy phenotype as determined by simultaneously the OCR and extracellular acidification rate (ECAR) from the same experiment, and non-mitochondrial respiration (**h**). *n*=4 in (**f-h**). **i**, Cortical brain sections from LRRK2-G2019S heterozygous knockin mice have increased F-actin as assessed by staining with fluorescent phalloidin and quantified in (**j**). Scale bar in (**i**) represents 75 µm. **k**, Native gel electrophoresis followed by western blotting for LRRK2 reveals an increased multimer to monomer ratio in cortical homogenates from LRRK2-G2019S heterozygous knockin mice, as quantified in (**l**). *n*=6 per genotype in (**i-l**). Data are represented as mean ± SD. *p<0.05, **p<0.01, ***p<0.005, Student’s t-test. Mice are 10-12 weeks old

Consistent with our results in *Drosophila*, previous studies have implicated LRRK2 in actin cytoskeletal dynamics in mammalian systems [49–51]. To confirm and extend these studies, we examined neurons derived from induced pluripotent stem cells (iPSC) heterozygous for the G2019S mutation and heterozygous LRRK2 G2019S knockin mice. Staining of cortical neurons derived from iPSC heterozygous for the G2019S mutation and isogenic controls (Fig. 6a) with phalloidin revealed increased F-actin (Fig. 6b,c) as well as frequent actin aggregates (Fig. 6b arrows, 6d, Supplementary Fig. 6a). When metabolic activity of G2019S heterozygous neurons was compared to that of isogenic control neurons using the Seahorse XFe96 Analyzer, a significant reduction in mitochondrial and non-mitochondrial respiration was observed (Fig. 6e-h). To determine if dysregulation of the actin cytoskeleton occurs in vivo in mammalian systems, we analyzed brain tissue from heterozygous LRRK2-G2019S knockin mice. At 10–12 weeks of age, we observed significantly increased in F-actin as determined by fluorescent phalloidin staining in cryosections of cortex (Fig. 6i,j). Actin rods were not apparent within neurons from these young animals. We additionally prepared homogenates from brains of the heterozygous knockin mice and performed native gel electrophoresis followed by western blotting for LRRK2. Quantitative analysis revealed significantly increased multimers in mice heterozygous for LRRK2-G2019S compared to littermate controls (Fig. 6k-l, Supplementary Fig. 6b). Given our findings with LRRK2 in human cells and mice, we assessed the ability of human LRRK2 expression to influence α-synuclein associated phenotypes in *Drosophila*. When we expressed human LRRK2 in flies also expressing human α-synuclein, we observed worsened locomotor deficits (Supplementary Fig. 7a) and enhanced neurodegeneration (Supplementary Fig. 7b-e). Expression of human LRRK2 did not alter levels of transgenic human α-synuclein (Supplementary Fig. 7f). These findings support conserved function between fly Lrrk and human LRRK2.

### Autophagy defects are downstream of Lrrk-mediated actin stabilization

**Fig. 7:**
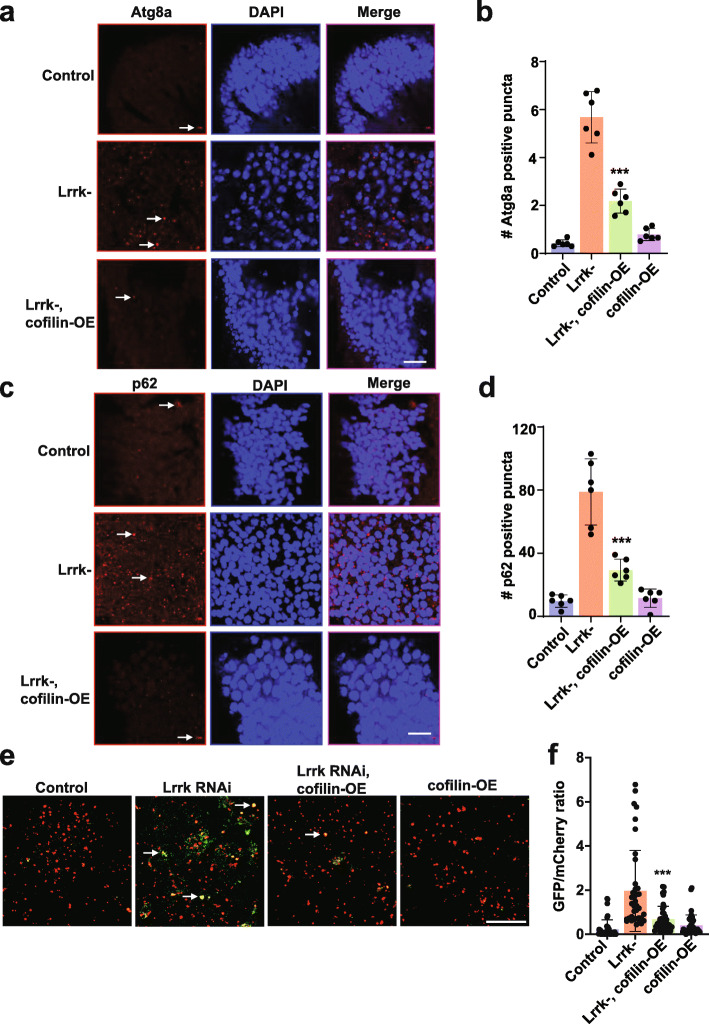
Autophagic pathology in Lrrk mutant flies is rescued by actin cytoskeletal destabilization.** a, **The number of Atg8a (LC3) immunoreactive puncta (arrows) is increased in the anterior medulla of homozygous Lrrk (*Lrrke*^03680^) mutant flies at 20 days of age. The increase in Lrrk mutants is reduced by expression of cofilin to destabilize the actin cytoskeleton, as quantified in (**b**). **c, **The number of p62-immunoreactive puncta (arrows) is increased in the medulla of homozygous Lrrk (*Lrrke*^03680^) mutant flies, and the increase in Lrrk mutants is reduced by expression of cofilin, as quantified in (**d**) when compared to control flies. Scale bars represent 10 µm in (**a,c**). *n*=6 per genotype. **e**, The number of GFP and mCherry puncta (arrows) is increased in flies with transgenic RNAi knockdown of Lrrk also expressing the GFP-mCherry-Atg8a reporter, and decreased by expression of cofilin. Scale bar represents 10 µm in (**e**). **f**, Quantification of the ratio of GFP to mCherry fluorescence indicates decreased autophagic flux in brains of flies with Lrrk knockdown mediated by transgenic RNAi, and partial normalization by overexpression of cofilin when compared to flies expressing transgenic Lrrk RNAi. *n*=4 per genotype. Data are represented as mean ± SD. ***p<0.005, ANOVA with Bonferroni post-test analysis. Control is *nSyb-GAL4/+ *in (**a-d**) and *UAS-GFP-mCherry-Atg8a/+; nSyb-GAL4/ +* in (**e,f**). Flies are 20 days old

A puzzling feature of LRRK2 biology and pathobiology has been the numerous cell biological pathways perturbed by manipulating LRRK2. Since the actin cytoskeleton controls many important cellular functions, these apparently divergent roles of LRRK2 might occur downstream effects of altered F-actin dynamics. To test this hypothesis directly, we examined autophagic pathology in Lrrk mutant flies. Autophagy, in particular, has been linked with LRRK2 function and dysfunction [16, 18, 22, 51, 52]. Homozygous protein null Lrrk (*Lrrke*^*03680*^) mutant flies appear relatively normal at the 10-day post eclosion time point used for our studies of modulation of α-synuclein (Figs. 1, 2 and 4) and tau[29] neurotoxicity. However, we have previously demonstrated excess stabilization of the actin cytoskeleton by 20 days of age with phalloidin precipitation and staining in *Lrrke*^*03680*^ mutants [29]. To determine if there was also evidence for dysregulated autophagy in older Lrrk mutants, we performed immunofluorescence for the autophagy-related gene 8a (Atg8a) protein in brains sections of flies aged to 20 days post-eclosion. Atg8a is the fly homolog of human LC3 and is widely used to mark autophagic structures in *Drosophila *[53]. We also performed immunostaining for the autophagic adaptor p62 (ref(2)P in *Drosophila*). Consistent with prior results in developing animals [18, 19], we observed evidence for altered autophagy in *Lrrke*^*03680*^ mutant flies as indicated by accumulation of Atg8a- and p62-immunoreactive puncta (Fig. 7a-d, arrows).

To examine autophagic flux directly, we expressed a GFP-mCherry-Atg8a reporter in neurons of flies with Lrrk knockdown mediated by transgenic RNAi. The tandem fluorescent GFP-mCherry-Atg8a protein can be used to follow the maturation progression of autolysosomes from autophagosomes as GFP fluorescence is quenched in the acidic environment of the autolysosome, leaving the protein with only the mCherry signal [33, 53–55]. The brains of Lrrk knockdown flies expressing the GFP-mCherry-Atg8a reporter displayed significantly increased numbers of green fluorescent puncta that were also positive for mCherry, assessed by quantifying the GFP/mCherry ratio (Fig. 7e arrows, f), consistent with autophagosomes. These findings are consistent with impaired autophagic flux following Lrrk knockdown in neurons.

To assess the role of abnormal F-actin stabilization in altered autophagy in Lrrk mutants, we overexpressed cofilin (twinstar in *Drosophila*), an actin binding and severing protein. We have previously demonstrated that cofilin overexpression reduces F-actin levels in transgenic fly brains [56]. Overexpression of cofilin reduced the number of Atg8a- and p62-immunoreactive puncta (Fig. 7a-d) in the brains of aged Lrrk mutant animals and partially normalized autophagic flux in Lrrk knockdown animals (Fig. 7e,f), consistent with a role for excess actin stabilization in promoting autophagic dysfunction caused by loss of Lrrk function.

## Discussion

Here we have used a *Drosophila* model of α-synucleinopathy and experiments in iPS cells and mice to define excessive stabilization of F-actin with consequent disruption of mitochondrial dynamics and function as a key common pathway perturbed by both α-synuclein and Lrrk/LRRK2. These findings fit well with human genetic data implicating both α-synuclein and LRRK2 in the pathogenesis of Parkinson’s disease, as well as with the neuropathological finding of frequent Lewy pathology (aggregated α-synuclein) in the brains of Parkinson’s disease patients with mutations in LRRK2 [57, 58]. Consistent with the human genetic data, multiple studies have suggested that LRRK2, particularly expression of LRRK2-G2019S, can modulate α-synuclein pathology in mouse and iPS cell models [59, 60]. However, the mechanisms implicated as underlying the interaction have been broad and include effects on endoplasmic reticulum stress [61], endocytosis [62], retromer function [63], autophagy [64, 65] and lysosomal degradation [66, 67]. Intriguingly, each of these pathways is controlled by the actin cytoskeleton, raising the possibility that LRRK2 may exert its influence via actin. We have directly tested a role for the actin cytoskeleton downstream of Lrrk in autophagy by genetically destabilizing the actin cytoskeleton and demonstrating that autophagic abnormalities are ameliorated in the brains of Lrrk mutant flies (Fig. 7). Consistent with a convergent effect of Lrrk and α-synuclein through the actin cytoskeleton, we have recently demonstrated a key role for the actin cytoskeleton in controlling autophagy and mitophagy in α-synuclein transgenic flies [68]. It will be important to test the role of perturbed actin dynamics in mediating the enhancement of α-synuclein neurotoxicity by Lrrk manipulation directly in future studies. Multiple other potential substrates and binding partners of LRRK2, including Rab proteins [21, 69], may contribute to regulation of autophagy and other cellular pathways perturbed by manipulation of LRRK2.

Our current and prior [29] observations that loss and gain of Lrrk/LRRK2 function alter F-actin stabilization in vivo (Figs. 2, 5 and 6) and in cell culture (Fig. 6) are broadly consistent with reports that the actin cytoskeleton is altered in LRRK2 models [49, 50, 70, 71]. However, the precise effects on F-actin levels and morphology have been diverse, perhaps reflecting the role of various implicated actin binding proteins including WAVE2 [70] and ezrin, radixin and moesin (ERM) family proteins [71]. Interestingly, a proteomic analysis of LRRK2 transfected cells revealed multiple actin-binding and actin cytoskeletal proteins as LRRK2 interacting proteins [72], suggesting that LRRK2 may regulate multiple aspects of actin cytoskeletal organization. The same study demonstrated that LRRK2 binds purified actin in vitro and promotes destabilization of F-actin, consistent with our data demonstrating that LRRK2 can directly sever actin filaments.

We additionally find that while monomeric and dimeric forms of LRRK2 effectively sever F-actin filaments in vitro, oligomeric forms of LRRK2 show no detectable actin severing activity (Fig. 3), suggesting a mechanism controlling the actin severing activity of LRRK, including in disease states. Multiple studies have emphasized the role of the GTPase domain in controlling dimerization of LRRK2 [44]. As a member of the ROCO family of G-proteins LRRK may be best classified as a G-protein activated by dimerization [15], with cycling of the protein back to the monomeric state following GTP hydrolysis. Although there is some variability in experimental results across the LRRK2 literature, most studies suggest that Parkinson’s disease-promoting mutations in the GTPase domain decrease the GTPase activity of LRRK2 and increase the proportion of GTP-bound LRRK2 [73]. Strikingly, the protective mutant R1398H has the opposite effect: increased GTPase activity and decreased GTP-bound LRRK2 [74]. Here we show that a mutation in fly Lrrk engineered to mimic the pathogenic R1441C mutation promotes the formation of higher order Lrrk species and enhances neurotoxicity in vivo, while a mutation designed to recapitulate the protective R1398H mutation decreases multimer formation and protects from α-synuclein neurotoxicity in vivo. These findings suggest that GTPase activity of LRRK is a key determinant of oligomerization and neurotoxicity. We also observe oligomerization of LRKK and enhanced neurotoxicity with the G2019S kinase activating mutant and the fly Lrrk variant designed to mimic this common human variant. Since dimerization and oligomerization of LRRK2 has been associated with increased kinase activity [75, 76], and reciprocally, kinase activity can influence GTPase activity [77], therapies targeting LRRK2 kinase activity [78] have the potential to exert a beneficial effect through modulation of oligomer formation and actin severing activity.

However, our data also suggest, consistent with multiple prior reports [18, 19, 22, 52, 79, 80], that strong loss of LRRK2 function can be deleterious, at least in the context of pathological levels of α-synuclein or advancing age [29]. Our in vivo (Figs. 1, 2, 4 and 6) and in vitro (Supplementary Fig. 1a,b) data specifically raise the possibility of a dominant negative effect of pathogenic LRRK2 variants. We present evidence that both Lrrk genetic loss of function and overexpression enhance the neurotoxicity of α-synuclein through the same mechanism of excess F-actin stabilization and mitochondrial dysfunction. We propose that decreased levels of LRRK elevate F-actin levels by reducing the levels of monomeric and dimeric LRRK species, which have actin severing activity (Fig. 3). Increased oligomerization of mixtures of wild type LRRK2 and disease-associated LRRK2 variants (Supplementary Fig. 1a,b), as would be present in heterozygous patients with Parkinson’s disease, together with the inability of LRRK2 oligomers to sever actin (Fig. 3) suggest a mechanism for a dominant negative effect. Increased levels of wild type LRRK may also act in a dominant negative fashion by promoting formation of higher order LRRK species (Fig. 3). Our findings are consistent with noncoding variation in LRRK2 that predisposes to Parkinson’s disease [81–83], presumably by altering levels of wild type LRRK2. Importantly, however, human genetic studies identifying LRRK2 variants with predicted loss of function effects [84] and reduced LRRK2 levels [85] in heterozygous individual in the absence of any evidence of a clinical movement disorder suggests that a safe therapeutic window may exist for LRRK2 inhibition.

Our finding of mitochondrial dysfunction controlled by excessive actin stabilization promoted by α-synuclein and LRRK dysfunction are consistent with prior findings in LRRK2-G2019S iPSC [86, 87] and more broadly with the strong implication of altered mitochondrial function in Parkinson’s disease [1]. Recessive mutations in the genes encoding parkin and PINK1 cause highly penetrant parkinsonism clinically similar to typical Parkinson’s disease. Parkin and PINK1 work in an epistatic pathway to control the turnover of damaged mitochondria through mitophagy [88]. Interestingly, parkin mutations appear to override LRRK2 mutations clinically [89], consistent with our data placing α-synuclein and Lrrk upstream of mitochondrial dynamics dysfunction through actin-mediated mislocalization of the key fission Drp1 (Fig. 4).

In fact, excessive stabilization of F-actin, modulation of F-actin levels by LRRK, and downstream disruption of mitochondrial dynamics are relevant to neurodegenerative disorders in addition to the α-synucleinopathies. We have previously demonstrated that direct binding of tau, a protein strongly implicated in the pathogenesis of Alzheimer’s disease and related tauopathies stabilizes F-actin, leading to a convergent downstream pathway of aberrant mitochondrial dynamics consequent to mislocalization of Drp1. Manipulation of LRRK in fly and mouse models of tauopathies further increases F-actin levels and promotes mitochondrial dysfunction and neurotoxicity [29]. These findings raise the intriguing possibility that a convergent cell biological pathway underlies the pathogenesis of the most common human neurodegenerative disorders, Alzheimer’s and Parkinson’s disease.

However, the enhancement of both α-synuclein and tau neurotoxicity by LRRK raises the possibility that the findings are not specific. The effects of Lrrk manipulation do not simply reflect sensitization of neurons to any type of cellular damage. We have previously demonstrated that altering Lrrk levels does not influence the toxicity of mutant SCA3, a polyglutamine-expanded protein linked to spinocerebellar ataxia type 3 (Machado-Joseph disease), in an unrelated *Drosophila* model of age-dependent neurodegeneration [29]. Nonetheless, the role of actin cytoskeletal abnormalities in a broader range of neurodegenerative disorders, and potential modulation by LRRK, requires further investigation.

Finally, our findings suggest possible new therapeutic approaches to Parkinson’s disease and related disorders. Despite the involvement of the actin cytoskeleton in many important cellular processes, there has been significant interest in developing actin-modulating therapies for oncology applications. More recent efforts have focused on actin regulatory proteins, including cofilin [90], to mitigate toxicity associate with general alterations of the actin cytoskeleton [91]. Modulation of LRRK2 GTPase activity represents another attractive drug target [44, 92]. Despite concerns regarding the effects of LRRK2 inhibitors on lung and kidney function [79], perhaps via autophagy dysregulation [22, 52], LRRK2 kinase inhibitors have acceptable safety profiles in nonhuman primates [93] and are currently in clinical trials in patients. Our results suggest that therapeutic modulation of LRRK2 GTPase activity may represent a viable strategy for normalization of the actin cytoskeleton and downstream effects on mitochondrial dynamics and autophagy.

## Conclusions

We demonstrate here that two key molecular players in Parkinson’s disease, α-synuclein and LRRK2, interact mechanistically via specific effects on the actin cytoskeleton. LRRK2 monomers and dimers, but not oligomers, sever actin in vitro. Disease-promoting LRRK2 mutations promote the formation of higher order LRRK species in vitro and in vivo, while a protective mutation reduces multimerization. In vivo, higher order multimers of LRRK are associated with increased actin stabilization and downstream mitochondrial morphological and functional abnormalities mediated by mislocalization of the Drp1 fission protein. These findings provide a molecular explanation for the interaction of two major proteins implicated in the pathogenesis of Parkinson’s disease and suggest potential new approaches for therapy development.

## Supplementary Information

**Additional file 1: Supplementary Figure 1.** LRRK2 oligomerization in vitro. a,b, Equimolar admixtures of recombinant human wild type and disease-linked mutant LRRK2 proteins show increased multimer formation compared with the same amount of wild type LRRK2 protein as visualized by silver staining (a, left) and western blotting (a, right) compared with equal total amounts of individual proteins following a one hour incubation in vitro, as quantified in (b). *n*=3. Data are represented as mean ± SD. *p<0.05, ANOVA with Bonferroni post-test analysis, compared to LRRK2 control. **Supplementary Figure 2.** Lrrk manipulation exacerbates α-synuclein induced mitochondrial defects but does not change Drp1 levels. a, Metabolic profiling of whole brains in Seahorse XF 96-well culture microplates (Agilent) reveals decreased ECAR, which is worsened by Lrrk manipulation. *n*=6-9 per genotype. b,c, Mitochondrial morphology and localization of the fission protein Drp1 (arrows) are abnormal in brain sections from a-synuclein transgenic flies, and worsened with manipulation of Lrrk, as reflected in measurement of Pearson’s correlation coefficient (c) when compared to flies expressing α-synuclein alone. Scale bar in (b) represents 2 µm. *n*=6 per genotype. d, Expression of Drp1 does not change with manipulation of α-synuclein or Lrrk as indicated by western blotting with an antibody to HA to detect tagged Drp1. The blot is reprobed for GAPDH to illustrate equivalent protein loading. Data are represented as mean ± SD. ***p<0.005, ANOVA with Bonferroni post-test analysis. Control is *nSyb-GAL4,nSybQF2/+ *in (a) and *UAS-mito-GFP/+;Drp1*^HA^/*nSyb-GAL4,nSybQF2/+* in (b-d). Flies are 10 days old in (a-c) and 1 day old in (d). **Supplementary Figure 3.** Expression of Lrrk-R1069C enhances α-synuclein neurotoxicity. a, Expression of Lrrk-R1069C (analogous to human LRRK2-R1441C) enhances the toxicity of human α-synuclein as shown by reduced climbing ability when compared to flies expressing α-synuclein alone. b-e, Lrrk-R1069C expression promotes loss of medullary hematoxylin-stained neurons (b), as quantified in (c), and tyrosine hydroxylase immunostained neurons (d arrows), as quantified in (e) when compared to flies expressing α-synuclein alone. Scale bars represent 10 µm in (b) and 5 µm in (d). *n*=6 per genotype. f, Western blotting reveals no change in transgenic human α-synuclein levels with expression of Lrrk-R1069C. The blot is reprobed for GAPDH to illustrate equivalent protein loading. g,h, Formation of actin rods (arrows) is increased by expression of Lrrk-R1069C, as quantified in (h) when compared to flies expressing α-synuclein alone. Scale bar in (g) represents 15 µm. i,j, The α-synuclein-induced decline in OCR is worsened by expression of Lrrk-R1069C when compared to flies expressing α-synuclein alone. k) Native gel electrophoresis followed by western blotting (k) and quantitative analysis (l) demonstrates a significant increase in the Lrrk multimer to monomer ratio in flies expressing Lrrk-R1069C. *n*=3 per genotype. Data are represented as mean ± SD. *p<0.05, **p<0.01, *** *p*<0.005, ANOVA with Bonferroni post-test analysis. Control is *nSyb-GAL4, nSybQF2/+ *in (a-j) and *nSyb-GAL4/+* in (k). Lrrk-HA is *Lrrk*^HA^, *nSyb-GAL4/+* in (k-l). Flies are 10 days old in all panels except (f), in which flies are 1 day old. **Supplementary Figure 4.** Lrrk expression and effects on actin dynamics. a, Quantitative real time PCR reveals similar levels of expression of Lrrk transgenes used in the study. b,c, Expression of Lrrk-Q1003H in the absence of human α-synuclein expression does not influence the levels of F-actin as assessed by staining with fluorescent phalloidin (b) or promote actin rod formation (c). Scale bars are 75 µm in (a) and 15 µm in (c). *n*=4. Data are represented as mean ± SD. ANOVA with Bonferroni post-test analysis was used for statistical analysis. Control is *nSyb-GAL4, nSybQF2/+. *Flies are 1 day old in (a) and 10 days old in (b,c). **Supplementary Figure 5.** Lrrk-3KD has reduced neurotoxicity. a, Expression of a mutant form of Lrrk (Lrrk-3KD) with impaired kinase activity has reduced ability to enhance the locomotor climbing defect induced by the expression of transgenic human wild type human α-synuclein. b, Lrrk-3KD expression only modestly enhances α-synuclein-mediated loss of hematoxylin-stained neurons in the anterior medulla, as quantified in (c) when compared to flies expressing α-synuclein alone. Scale bar represents 10 µm in (b). d, Lrrk-3KD expression only modestly enhances the α-synuclein-induced loss of tyrosine hydroxylase-positive neurons in the anterior medulla (arrows), as quantified in (e) when compared to flies expressing α-synuclein alone. Scale bar represents 5µm in (d). f, Western blotting reveals no change in α-synuclein levels with expression of Lrrk-3KD. The blot is reprobed for GAPDH to illustrate equivalent protein loading. g, The number of actin-immunoreactive rods is only modestly increased when Lrrk-3KD is expressed (arrows), as quantified in (h) when compared to flies expressing α-synuclein alone. Scale bar in (g) represents 15 µm. i,j, The α-synuclein induced decline in OCR is not significantly changed by expression of Lrrk-3KD when compared to flies expressing α-synuclein alone. k, Native gel electrophoresis followed by western blotting (k) and quantitative analysis (l) demonstrates no change in the ratio of Lrrk multimer to monomer in flies expressing Lrrk-3KD compared to Lrrk-HA control flies. *n*=3-6 per genotype. Data are represented as mean ± SD. *p<0.05, **p<0.01, ***p<0.005, ANOVA with Bonferroni post-test analysis. Control is *nSyb-GAL4, nSybQF2/+ *in (a-j) and *nSyb-GAL4/+* in (k). Lrrk-HA is *Lrrk*^HA^, *nSyb-GAL4/+* in (k,l). Flies are 10 days old in (a-d, g-l) and 1 day old in (f). **Supplementary Figure 6.** Actin aggregates are present in human neurons heterozygous for LRRK2-G2019S. a, Phalloidin staining highlights actin aggregates (arrows) in iPSC-derived neurons carrying the PD-related LRRK2-G2019S mutation in the heterozygous state. The image in (a) is presented at a lower intensity compared to Fig. 6b to demonstrate aggregate morphology. b, Native gel electrophoresis followed by western blot analysis on wild type (+/+) and LRRK2 knockout (-/-) mice demonstrates the specificity of the anti-LRRK2 antibody used to detect oligomers in vivo*.* **SupplementaryFigure 7**. Expression of human LRRK2 enhances α-synuclein neurotoxicity. a, Expression of human LRRK2 enhances the locomotor climbing defect induced by the expression of transgenic human wild type human α-synuclein. b, Human LRRK2 expression enhances α-synuclein-mediated loss of hematoxylin-stained neurons in the anterior medulla, as quantified in (c) when compared to flies expressing α-synuclein alone. Scale bar represents 10 µm in (b). d, Human LRRK2 expression enhances the α-synuclein-induced loss of tyrosine hydroxylase-positive neurons in the anterior medulla (arrows), as quantified in (e) when compared to flies expressing α-synuclein alone. Scale bar represents 5 µm in (d). f, Western blotting reveals no change in α-synuclein levels with expression of human LRRK2. The blot is reprobed for GAPDH to illustrate equivalent protein loading. *n*=3-6 per genotype. Data are represented as mean ± SD. ***p<0.005, ANOVA with Bonferroni post-test analysis. Control is *nSyb-GAL4, nSybQF2/+*. Flies are 10 days old in (a-e) and 1 day old in (f).

## Data Availability

All data and materials are available upon request to authors.

## Abbreviations

LRRK2: Leucine-rich repeat kinase 2
Roc: Ras of complex proteins
COR: C terminal of Ro
