# Supplementary material for: Oligomerization of Lrrk controls actin severing and α-synuclein neurotoxicity in vivo
Source: Mol Neurodegener. 2021 May 24;16:33. doi: 10.1186/s13024-021-00454-3 (PMC8142648; doi:10.1186/s13024-021-00454-3)
Supplement: Supplementary file 1 — Additional file 1: Supplementary Figure 1. LRRK2 oligomerization in vitro. a,b, Equimolar admixtures of recombinant human wild type and disease-linked mutant LRRK2 proteins show increased multimer formation compared with the same amount of wild type LRRK2 protein as visualized by silver staining (a, left) and western blotting (a, right) compared with equal total amounts of individual proteins following a one hour incubation in vitro, as quantified in (b). n=3. Data are represented as mean ± SD. *p<0.05, ANOVA with Bonferroni post-test analysis, compared to LRRK2 control. Supplementary Figure 2. Lrrk manipulation exacerbates α-synuclein induced mitochondrial defects but does not change Drp1 levels. a, Metabolic profiling of whole brains in Seahorse XF 96-well culture microplates (Agilent) reveals decreased ECAR, which is worsened by Lrrk manipulation. n=6-9 per genotype. b,c, Mitochondrial morphology and localization of the fission protein Drp1 (arrows) are abnormal in brain sections from a-synuclein transgenic flies, and worsened with manipulation of Lrrk, as reflected in measurement of Pearson’s correlation coefficient (c) when compared to flies expressing α-synuclein alone. Scale bar in (b) represents 2 µm. n=6 per genotype. d, Expression of Drp1 does not change with manipulation of α-synuclein or Lrrk as indicated by western blotting with an antibody to HA to detect tagged Drp1. The blot is reprobed for GAPDH to illustrate equivalent protein loading. Data are represented as mean ± SD. ***p<0.005, ANOVA with Bonferroni post-test analysis. Control is nSyb-GAL4,nSybQF2/+ in (a) and UAS-mito-GFP/+;Drp1HA/nSyb-GAL4,nSybQF2/+ in (b-d). Flies are 10 days old in (a-c) and 1 day old in (d). Supplementary Figure 3. Expression of Lrrk-R1069C enhances α-synuclein neurotoxicity. a, Expression of Lrrk-R1069C (analogous to human LRRK2-R1441C) enhances the toxicity of human α-synuclein as shown by reduced climbing ability when compared to flies expressing α-synuclein alone. b-e, [file 13024_2021_454_MOESM1_ESM.pdf]

Supplementary Fig. 1

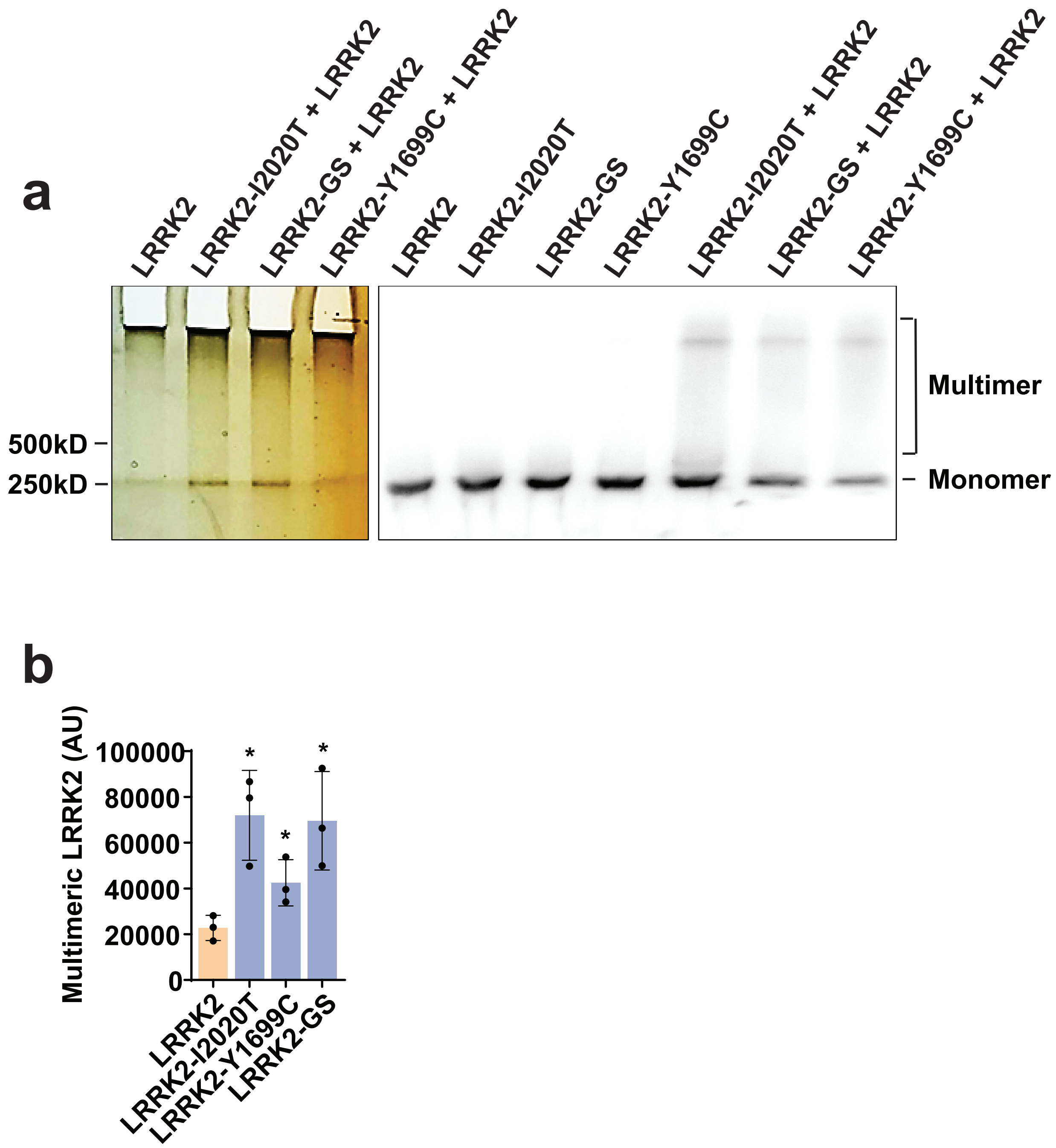

Supplementary Fig. 1. LRRK2 oligomerization in vitro. a,b, Equimolar admixtures of recombinant human wild type and disease-linked mutant LRRK2 proteins show increased multimer formation compared with the same amount of wild type LRRK2 protein as visualized by silver staining (**a**, left) and western blotting (**a**, right) compared with equal total amounts of individual proteins following a one hour incubation in vitro, as quantified in (**b**). n=3. Data are represented as mean ± SD. *p<0.05, ANOVA with Bonferroni post-test analysis, compared to LRRK2 control.

Supplementary Fig. 2

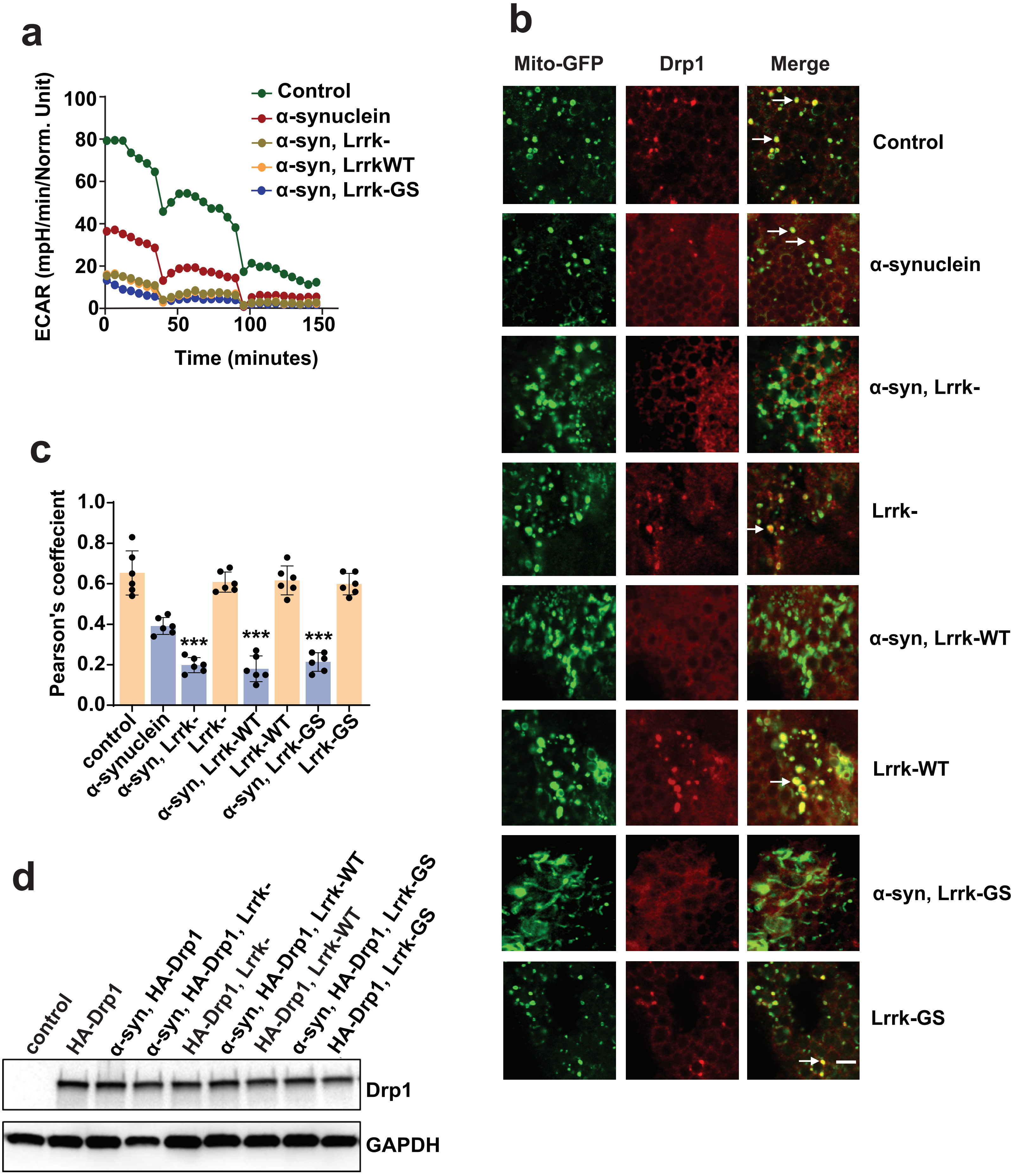

Supplementary Fig. 2. Lrrk manipulation exacerbates α-synuclein induced mitochondrial defects but does not change Drp1 levels. a, Metabolic profiling of whole brains in Seahorse XF96-well culture microplates (Agilent) reveals decreased ECAR, which is worsened by Lrrk manipulation. n=6-9 per genotype. **b,c**, Mitochondrial morphology and localization of the fission protein Drp1 (arrows) are abnormal in brain sections from α-synuclein transgenic flies, and worsened with manipulation of Lrrk, as reflected in measurement of Pearson's correlation coefficient (**c**) when compared to flies expressing **α**-synuclein alone. Scale bar in (**b**) represents 2 μm. n=6 per genotype. **d**, Expression of Drp1 does not change with manipulation of **α**-synuclein or Lrrk as indicated by western blotting with an antibody to HA to detect tagged Drp1. The blot is reprobed for GAPDH to illustrate equivalent protein loading. Data are represented as mean ± SD. ***p<0.005, ANOVA with Bonferroni post-test analysis. Control is *nSyb-GAL4, nSybQF2/+* in (**a**) and *UAS-mito-GFP/+; Drp1*^{HA}*/ nSyb-GAL4, nSybQF2/+* in (**b-d**). Flies are 10 days old in (**a-c**) and 1 day old in (**d**).

Supplementary Fig. 3

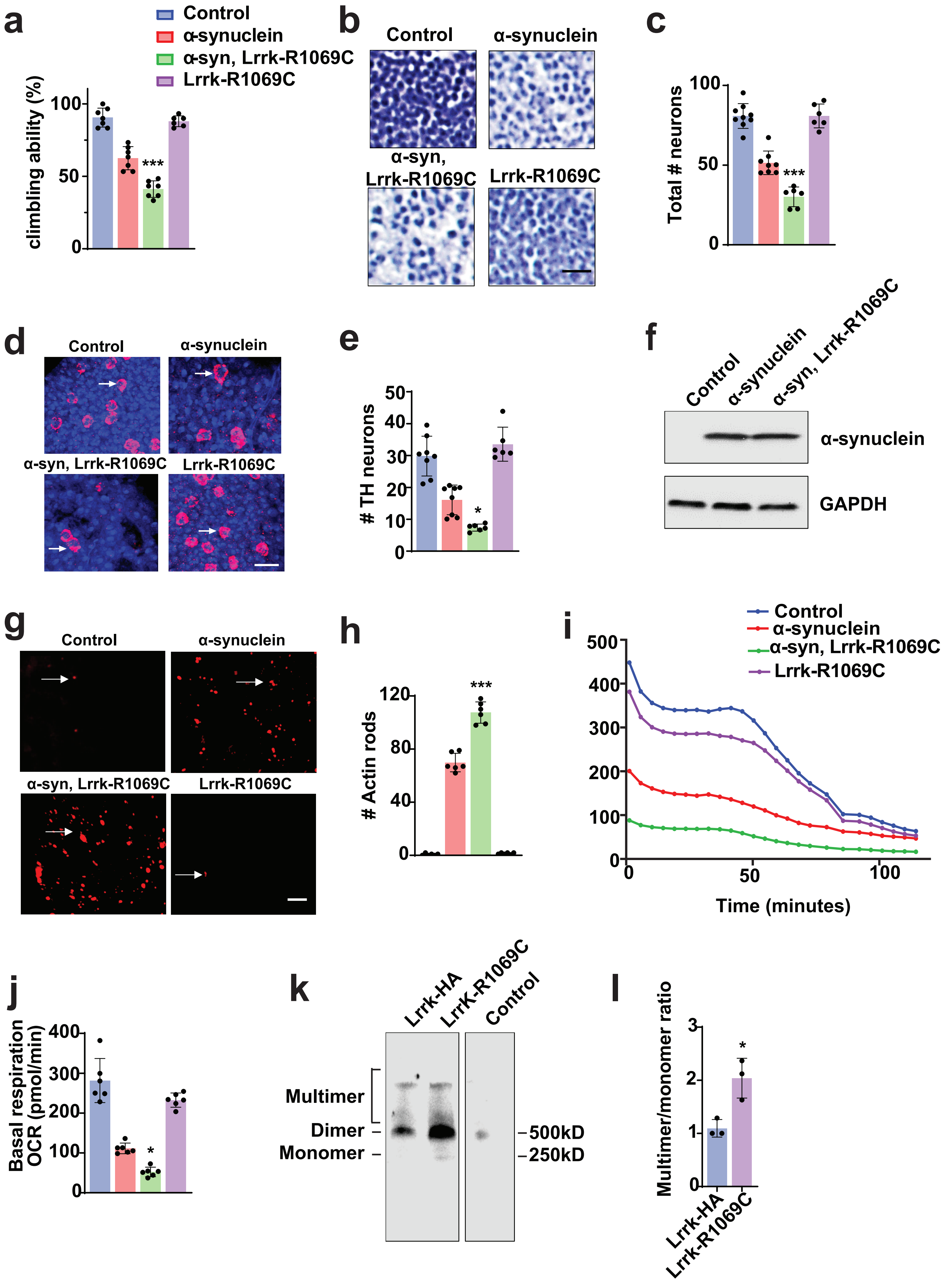

Supplementary Fig. 3. Expression of Lrrk-R1069C enhances α-synuclein neurotoxicity. a, Expression of Lrrk-R1069C (analogous to human LRRK2-R1441C) enhances the toxicity of human α-synuclein as shown by reduced climbing ability when compared to flies expressing α-synuclein alone. **b-e**, Lrrk-R1069C expression promotes loss of medullary hematoxylin-stained neurons (**b**), as quantified in (**c**), and tyrosine hydroxylase immunostained neurons (**d** arrows), as quantified in (**e**) when compared to flies expressing α-synuclein alone. Scale bars represent 10 μm in (**b**) and 5 μm in (**d**). n=6 per genotype. **f**, Western blotting reveals no change in transgenic human α-synuclein levels with expression of Lrrk-R1069C. The blot is reprobed for GAPDH to illustrate equivalent protein loading. **g,h,** Formation of actin rods (arrows) is increased by expression of Lrrk-R1069C, as quantified in (**h**) when compared to flies expressing α-synuclein alone. Scale bar in (**g**) represents 15 μm. **i,j**, The α-synuclein-induced decline in OCR is worsened by expression of Lrrk-R1069C when compared to flies expressing α-synuclein alone. **k**) Native gel electrophoresis followed by western blotting (**k**) and quantitative analysis (**l**) demonstrates a significant increase in the Lrrk multimer to monomer ratio in flies expressing Lrrk-R1069C. n=3 per genotype. Data are represented as mean ± SD. *p<0.05, ** p<0.01, *** p<0.005, ANOVA with Bonferroni post-test analysis. Control is *nSyb-GAL4, nSybQF2/+* in (a-j) and *nSyb-*

GAL4/+ in (**k**). Lrrk-HA is *Lrrk*^{HA}, *nSyb-GAL4/+* in (**k-l**). Flies are 10 days old in all panels except (**f**), in which flies are 1 day old.

Supplementary Fig. 4

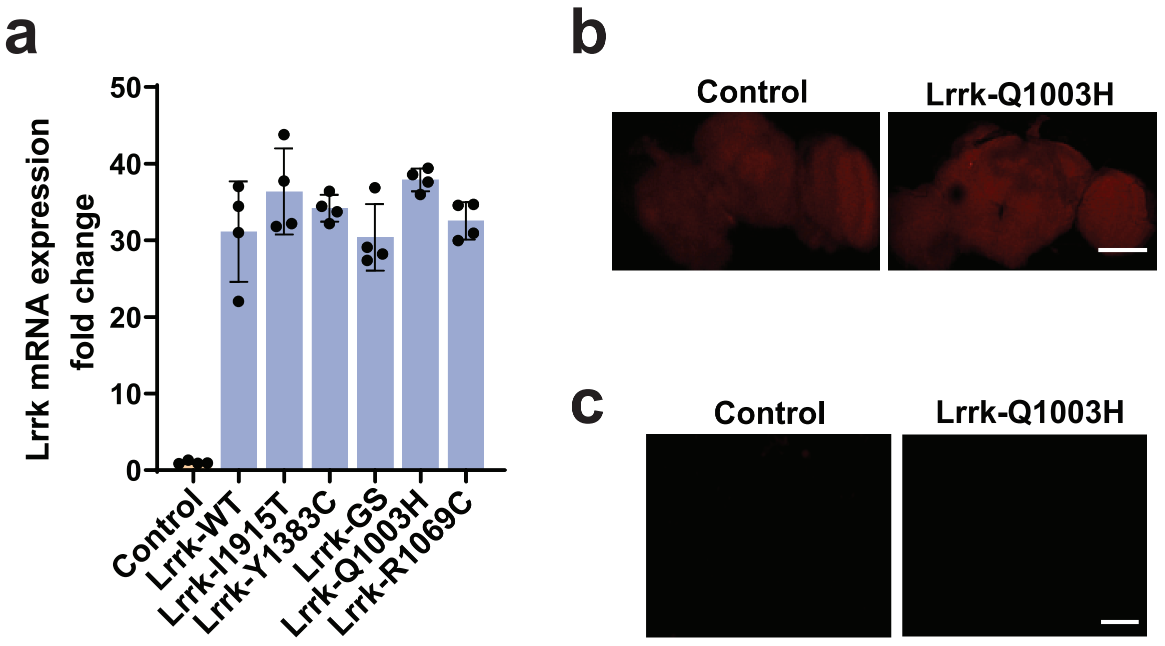

Supplementary Fig. 4. Lrrk expression and effects on actin dynamics. a, Quantitative real time PCR reveals similar levels of expression of Lrrk transgenes used in the study. **b,c**, Expression of Lrrk-Q1003H in the absence of human **α**-synuclein expression does not influence the levels of F-actin as assessed by staining with fluorescent phalloidin (**b**) or promote actin rod formation (**c**). Scale bars are 75 μm in (**a**) and 15 μm in (**c**). n=4. Data are represented as mean ± SD. ANOVA with Bonferroni post-test analysis was used for statistical analysis. Control is *nSyb-GAL4, nSybQF2/+*. Flies are 1 day old in (**a**) and 10 days old in (**b,c**).

Supplementary Fig. 5

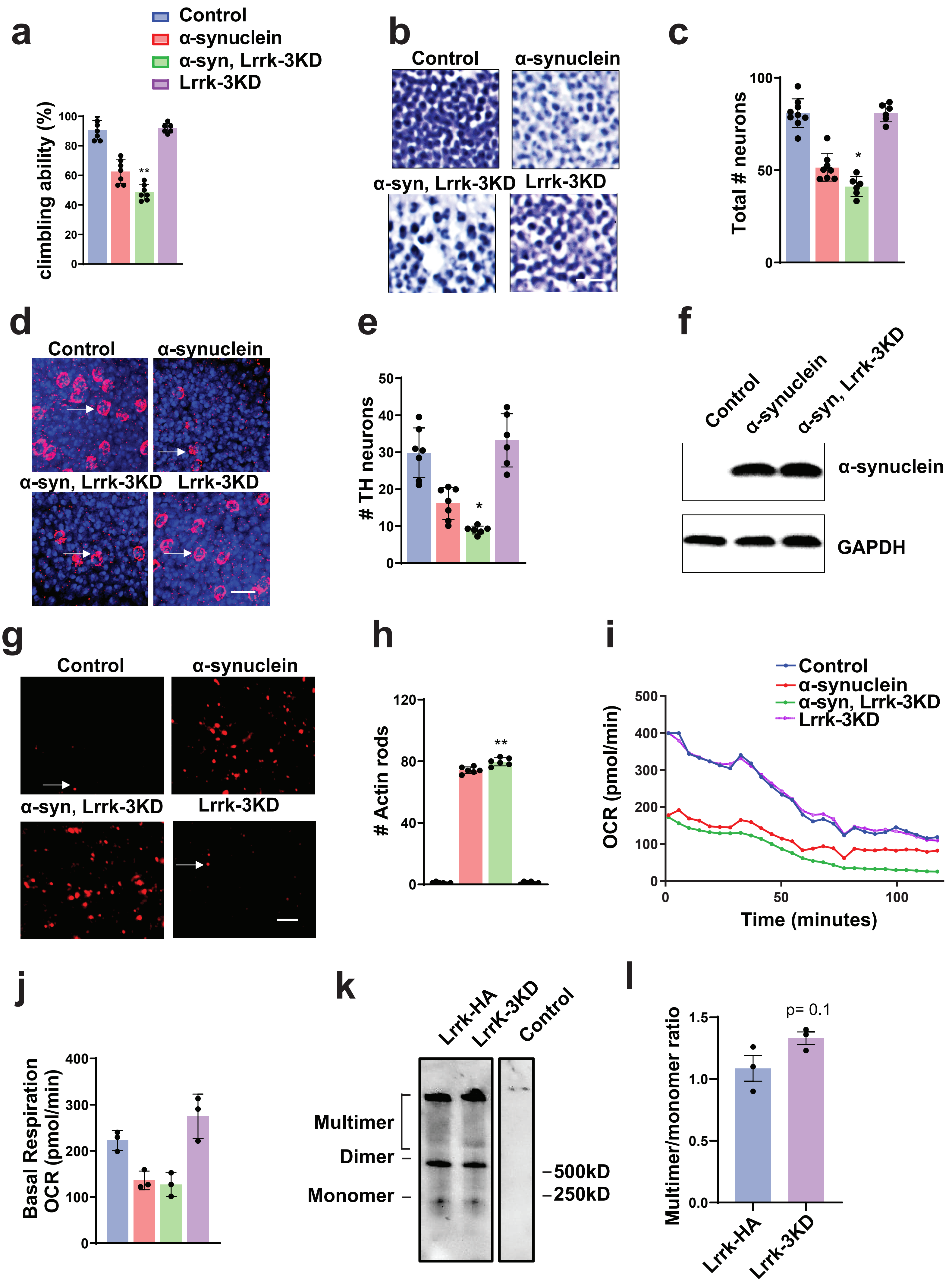

Supplementary Fig. 5. Lrrk-3KD has reduced neurotoxicity. a, Expression of a mutant form of Lrrk (Lrrk-3KD) with impaired kinase activity has reduced ability to enhance the locomotor climbing defect induced by the expression of transgenic human wild type human **α**-synuclein. **b**, Lrrk-3KD expression only modestly enhances **α**-synuclein-mediated loss of hematoxylin-stained neurons in the anterior medulla, as quantified as quantified in (**c**) when compared to flies expressing **α**-synuclein alone. Scale bar represents 10 μm in (**b**). **d**, Lrrk-3KD expression only modestly enhances the **α**-synuclein-induced loss of tyrosine hydroxylase-positive neurons in the anterior medulla (arrows), as quantified in (**e**) when compared to flies expressing **α**-synuclein alone. Scale bar represents 5 μm in (**d**). **f**, Western blotting reveals no change in **α**-synuclein levels with expression of Lrrk-3KD. The blot is reprobed for GAPDH to illustrate equivalent protein loading. **g**, The number of actin-immunoreactive rods is only modestly increased when Lrrk-3KD is expressed (arrows), as quantified in (**h**) when compared to flies expressing **α**-synuclein alone. Scale bar in (**g**) represents 15 μm. **i,j**, The **α**-synuclein induced decline in OCR is not significantly changed by expression of Lrrk-3KD when compared to flies expressing **α**-synuclein alone. **k**, Native gel electrophoresis followed by western blotting (**k**) and quantitative analysis (**l**) demonstrates no change in the ratio of Lrrk multimer to monomer in flies expressing Lrrk-3KD compared to Lrrk-HA control flies. n=3-6 per genotype. Data

are represented as mean ± SD. *p<0.05, **p<0.01, ***p<0.005, ANOVA with Bonferroni post-test analysis. Control is *nSyb-GAL4, nSybQF2/+* in (**a-j)** and *nSyb-GAL4/+* in (**k**). Lrrk-HA is *Lrrk^{HA}*, *nSyb-GAL4/+* in (**k,l**). Flies are 10 days old in (**a-d, g-l**) and 1 day old in (**f**).

Supplementary Fig. 6

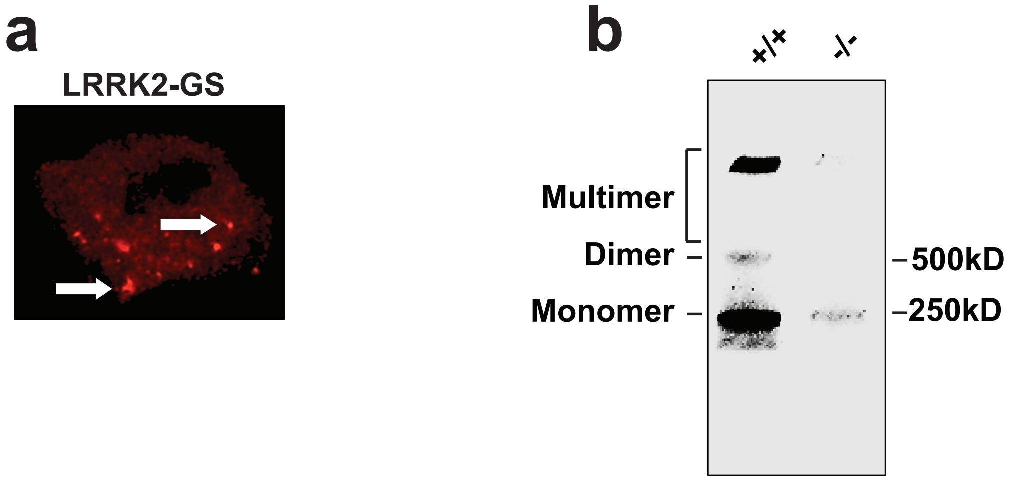

Supplementary Fig. 6. Actin aggregates are present in human neurons heterozygous for LRRK2-G2019S. a, Phalloidin staining highlights actin aggregates (arrows) in iPSC-derived neurons carrying the PD-related LRRK2-G2019S mutation in the heterozygous state. The image in (**a**) is presented at a lower intensity compared to Fig. 6b to demonstrate aggregate morphology. **b**, Native gel electrophoresis followed by western blot analysis on wild type (+/+) and LRRK2 knockout (-/-) mice demonstrates the specificity of the anti-LRRK2 antibody used to detect oligomers in vivo.

Supplementary Fig. 7

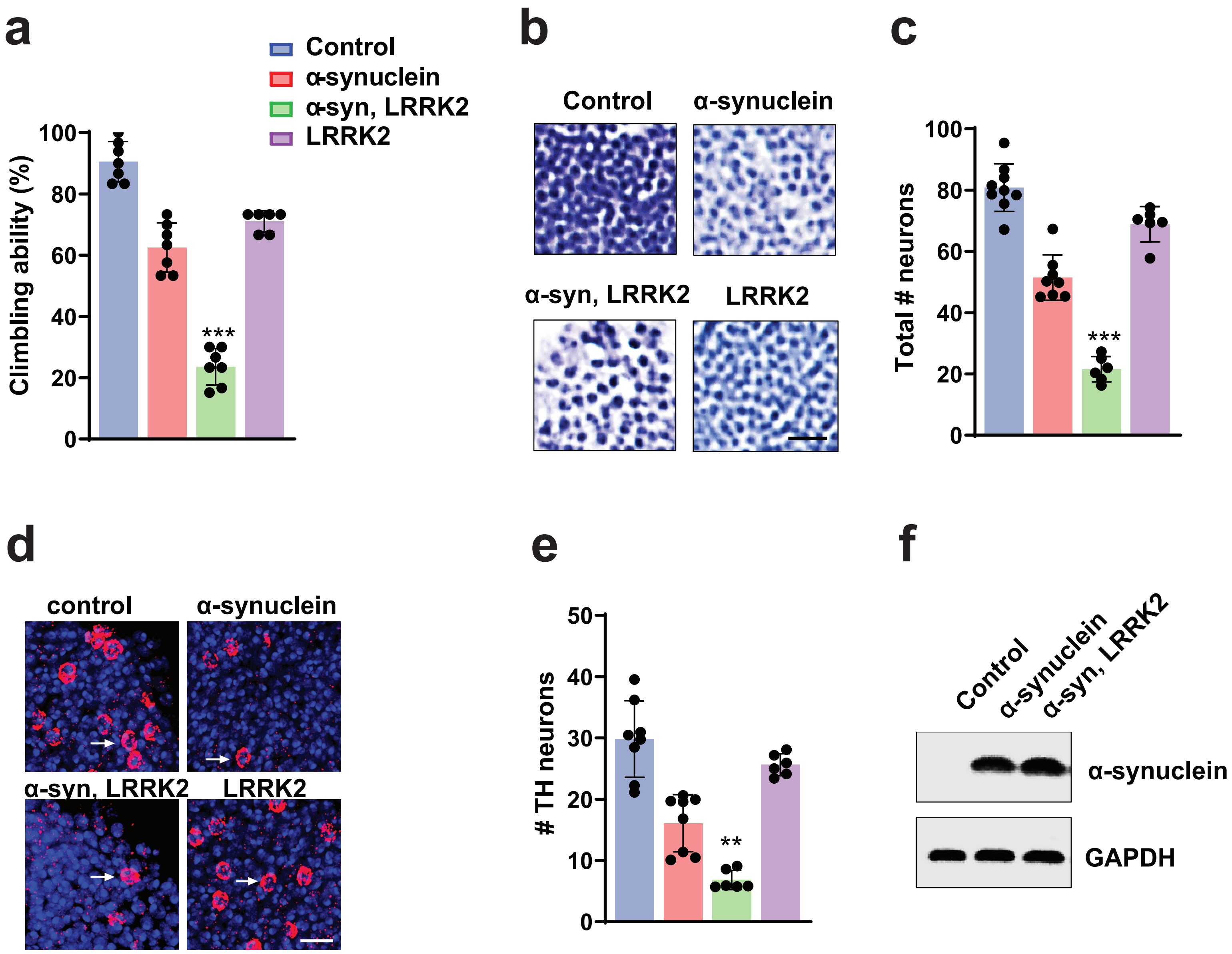

Supplementary Fig. 7. Expression of human LRRK2 enhances α-synuclein neurotoxicity. a, Expression of human LRRK2 enhances the locomotor climbing defect induced by the expression of transgenic human wild type human **α**-synuclein. **b**, Human LRRK2 expression enhances **α**-synuclein-mediated loss of hematoxylin-stained neurons in the anterior medulla, as quantified as quantified in (**c**) when compared to flies expressing **α**-synuclein alone. Scale bar represents 10 μm in (**b**). **d**, Human LRRK2 expression enhances the **α**-synuclein-induced loss of tyrosine hydroxylase-positive neurons in the anterior medulla (arrows), as quantified in (**e**) when compared to flies expressing **α**-synuclein alone. Scale bar represents 5 μm in (**d**). **f**, Western blotting reveals no change in **α**-synuclein levels with expression of human LRRK2. The blot is reprobed for GAPDH to illustrate equivalent protein loading. n=3-6 per genotype. Data are represented as mean ± SD. ***p<0.005, ANOVA with Bonferroni post-test analysis. Control is *nSyb-GAL4, nSybQF2/+*. Flies are 10 days old in (**a-e**) and 1 day old in (**f**).
